# Cytokine-responsive T- and NK-cells portray SARS-CoV-2 vaccine-responders and infection in multiple myeloma patients

**DOI:** 10.1038/s41375-023-02070-0

**Published:** 2023-12-04

**Authors:** Julius C. Enssle, Julia Campe, Alina Moter, Isabel Voit, Alec Gessner, Weijia Yu, Sebastian Wolf, Björn Steffen, Hubert Serve, Melanie Bremm, Sabine Huenecke, Michael Lohoff, Maria Vehreschild, Holger F. Rabenau, Marek Widera, Sandra Ciesek, Thomas Oellerich, Katharina Imkeller, Michael A. Rieger, Ivana von Metzler, Evelyn Ullrich

**Affiliations:** 1 Goethe University Frankfurt, University Hospital, Department of Medicine II - Hematology and Oncology, Frankfurt am Main, Germany; 2 Frankfurt Cancer Institute (FCI), Frankfurt am Main, Germany; 3 German Cancer Consortium (DKTK), partner site Frankfurt/Mainz, a partnership between DKFZ and University Hospital Frankfurt, Frankfurt am Main, Germany; 4 Goethe University Frankfurt, Department of Pediatrics, Experimental Immunology and Cell Therapy, Frankfurt am Main, Germany; 5 Goethe University Frankfurt, University Hospital, Department of Pediatrics, Frankfurt am Main, Germany; 6 Institute of Medical Microbiology and Hospital Hygiene, Philipps University, Marburg, Germany; 7 Goethe University Frankfurt, University Hospital, Department of Medicine II - Infectious Diseases, Frankfurt am Main, Germany; 8 Goethe University Frankfurt, University Hospital, Institute for Medical Virology, Frankfurt am Main, Germany; 9 German Centre for Infection Research, external partner site, Frankfurt am Main, Germany; 10 Fraunhofer Institute for Translational Medicine and Pharmacology ITMP, Frankfurt am Main, Germany; 11 Goethe University Frankfurt, University Hospital, Edinger Institute (Neurological Institute), Frankfurt am Main, Germany; 12 Goethe University Frankfurt, University Hospital, MSNZ Group of Computational Immunology, Frankfurt am Main, Germany; 13 University Cancer Center (UCT), Frankfurt am Main, Germany; 14 Cardio-Pulmonary Institute, Frankfurt am Main, Germany

**Keywords:** Translational research, Infectious diseases, Myeloma, Infectious diseases

## Abstract

Patients with multiple myeloma (MM) routinely receive mRNA-based vaccines to reduce COVID-19-related mortality. However, whether disease- and therapy-related alterations in immune cells and cytokine-responsiveness contribute to the observed heterogeneous vaccination responses is unclear. Thus, we analyzed peripheral blood mononuclear cells from patients with MM during and after SARS-CoV-2 vaccination and breakthrough infection (BTI) using combined whole-transcriptome and surface proteome single-cell profiling with functional serological and T-cell validation in 58 MM patients. Our results demonstrate that vaccine-responders showed a significant overrepresentation of cytotoxic CD4^+^ T- and mature CD38^+^ NK-cells expressing FAS^+^/TIM3^+^ with a robust cytokine-responsiveness, such as type-I-interferon-, IL-12- and TNF-α-mediated signaling. Patients with MM experiencing BTI developed strong serological and cellular responses and exhibited similar cytokine-responsive immune cell patterns as vaccine-responders. This study can expand our understanding of molecular and cellular patterns associated with immunization responses and may benefit the design of improved vaccination strategies in immunocompromised patients.

## Introduction

During the COVID-19 pandemic, patients with multiple myeloma (MM) were among the first to receive mRNA-based SARS-CoV-2 vaccines [1]. Immunological responses to SARS-CoV-2 vaccination and breakthrough infection (BTI) in patients with hematological malignancies have been extensively analyzed [2–8]. Patients with MM exhibited heterogeneous serological and T-cell vaccination responses against SARS-CoV-2 [9–14]. Sufficient immune response is associated with strong neutralizing antibodies with high avidity in MM patients [15]. However, SARS-CoV-2 BTI is associated with mortality, even in fully vaccinated MM patients [16]. Therefore, characterizing and understanding the factors and mechanisms associated with vaccination failure is crucial.

Single-cell sequencing technologies facilitate characterizing cellular and molecular immune responses, particularly in severe COVID-19 [17–20]. Patients with hematologic malignancies have a highly heterogeneous response to SARS-CoV-2, likely owing to disease- and therapy-related alterations in peripheral immunity [13]. However, in-depth characterization of the peripheral immune cell compartment in patients with cancer and immunosuppression in context of SARS-CoV-2 infection and COVID-19 vaccination is limited.

This study elucidated the underlying immune regulation by analyzing peripheral blood mononuclear cell (PBMC)-derived B-, T-, NK- and NKT-cells from a cohort of MM patients and age-matched healthy controls during and after SARS-CoV-2 vaccination and BTI via combined whole-transcriptome and surface proteome single-cell sequencing analysis. Overall, we believe that these findings not only help designing improved and variant-adapted vaccination strategies for MM patients, but might be transferrable to immunocompromised patients with impairment of B-cell function in general.

## Materials and methods

### Study design

This retrospective, non-interventional and observatory study aimed at investigating the response of patients with MM to SARS-CoV-2 vaccination and BTI in a real-world perspective combining single-cell profiling and functional evaluation of relevant immune cell populations. The entire observation cohort of all patients with MM treated at our institution comprised of 105 patients (Table S1). For detailed follow-up analysis, 58 patients with MM and present residual material for the individual immune response assays were included who were treated at our institution between January 2021 to February 2023 (Table 1). All individuals declared written informed consent and the study was approved by the local ethics committee Frankfurt, Germany (Ethics vote number: UCT-5-2021). Detailed information on the sample/clinical data acquisition and vaccination scheme/history are depicted in the Supplementary material.

**Table 1:** Patient characteristics of the long-term study cohort.

	Overall	No infection	Pre 3rd	Post 3rd	*p*-value
N patients, *n* (%)	58 (100.0)	17 (29.3)	14 (24.1)	27 (46.6)	
Female sex, *n* (%)	28 (48.3)	10 (58.8)	5 (35.7)	13 (48.1)	0.44
Age, median [IQR]	65.00 [57.25, 72.00]	66.00 [64.00, 78.00]	62.00 [53.50, 72.00]	64.00 [56.50, 69.00]	0.187
Type of MM, *n* (%)					0.657
IgG	1 (1.7)	1 (5.9)	0 (0.0)	0 (0.0)	
IgA	30 (51.7)	9 (52.9)	7 (50.0)	14 (51.9)	
LC	13 (22.4)	3 (17.6)	3 (21.4)	7 (25.9)	
Smouldering	13 (22.4)	4 (23.5)	3 (21.4)	6 (22.2)	
Asecretoric	1 (1.7)	0 (0.0)	1 (7.1)	0 (0.0)	
ISS, *n* (%)					0.273
1	23 (39.7)	6 (35.3)	3 (21.4)	14 (51.9)	
2	14 (24.1)	5 (29.4)	4 (28.6)	5 (18.5)	
3	15 (25.9)	6 (35.3)	4 (28.6)	5 (18.5)	
NA	6 (10.3)	0 (0.0)	3 (21.4)	3 (11.1)	
Revised ISS, *n* (%)					0.844
1	18 (31.0)	6 (35.3)	2 (14.3)	10 (37.0)	
2	23 (39.7)	6 (35.3)	7 (50.0)	10 (37.0)	
3	6 (10.3)	2 (11.8)	2 (14.3)	2 (7.4)	
NA	11 (19.0)	3 (17.6)	3 (21.4)	5 (18.5)	
Genomic high risk, *n* (%)					0.918
No	31 (53.4)	9 (52.9)	8 (57.1)	14 (51.9)	
Yes	17 (29.3)	4 (23.5)	4 (28.6)	9 (33.3)	
NA	10 (17.2)	4 (23.5)	2 (14.3)	4 (14.8)	
Current therapy line at TP5, *n* (%)					0.15
0	1 (1.8)	0 (0.0)	1 (7.7)	0 (0.0)	
1	35 (61.4)	6 (35.3)	9 (69.2)	20 (74.1)	
2	7 (12.3)	4 (23.5)	1 (7.7)	2 (7.4)	
3	6 (10.5)	4 (23.5)	0 (0.0)	2 (7.4)	
4	6 (10.5)	2 (11.8)	2 (15.4)	2 (7.4)	
5	1 (1.8)	1 (5.9)	0 (0.0)	0 (0.0)	
9	1 (1.8)	0 (0.0)	0 (0.0)	1 (3.7)	
Remission state at TP5, *n* (%)					0.134
CR/VGPR	43 (74.1)	11 (64.7)	10 (71.4)	22 (81.5)	
PR	7 (12.1)	4 (23.5)	0 (0.0)	3 (11.1)	
SD/PD	8 (13.8)	2 (11.8)	4 (28.6)	2 (7.4)	
Number of HDCT, *n* (%)					0.212
0	16 (27.6)	8 (47.1)	4 (28.6)	4 (14.8)	
1	27 (46.6)	5 (29.4)	8 (57.1)	14 (51.9)	
2	13 (22.4)	4 (23.5)	2 (14.3)	7 (25.9)	
3	2 (3.4)	0 (0.0)	0 (0.0)	2 (7.4)	
Time since last HDCT at TP5, months [IQR]	32.00 [17.00, 54.25]	24.00 [17.00, 71.00]	32.00 [22.25, 50.75]	36.00 [17.00, 47.50]	0.961
Therapy status at TP5, *n* (%)					0.012
Active therapy	22 (37.9)	12 (70.6)	2 (14.3)	8 (29.6)	
Maintenance	22 (37.9)	2 (11.8)	7 (50.0)	13 (48.1)	
No therapy	14 (24.1)	3 (17.6)	5 (35.7)	6 (22.2)	
Current imid-based therapy at TP5, *n* (%)	37 (63.8)	11 (64.7)	9 (64.3)	17 (63.0)	0.992
Current PI-based therapy at TP5 *n* (%)	8 (13.8)	3 (17.6)	1 (7.1)	4 (14.8)	0.685
Current antiCD38-based therapy at TP5, *n* (%)	15 (25.9)	6 (35.3)	3 (21.4)	6 (22.2)	0.572
Current therapy line at TP6, *n* (%)					0.176
0	1 (1.7)	0 (0.0)	1 (7.1)	0 (0.0)	
1	34 (58.6)	6 (35.3)	8 (57.1)	20 (74.1)	
2	6 (10.3)	3 (17.6)	1 (7.1)	2 (7.4)	
3	5 (8.6)	3 (17.6)	1 (7.1)	1 (3.7)	
4	8 (13.8)	4 (23.5)	1 (7.1)	3 (11.1)	
5	1 (1.7)	0 (0.0)	1 (7.1)	0 (0.0)	
7	1 (1.7)	1 (5.9)	0 (0.0)	0 (0.0)	
8	1 (1.7)	0 (0.0)	1 (7.1)	0 (0.0)	
9	1 (1.7)	0 (0.0)	0 (0.0)	1 (3.7)	
Remission state at TP6, *n* (%)					0.071
CR/VGPR	40 (69.0)	8 (47.1)	10 (71.4)	22 (81.5)	
PR	6 (10.3)	3 (17.6)	0 (0.0)	3 (11.1)	
SD/PD	12 (20.7)	6 (35.3)	4 (28.6)	2 (7.4)	
Therapy status at TP6, *n* (%)					0.082
Active therapy	25 (43.1)	12 (70.6)	4 (28.6)	9 (33.3)	
Maintenance	20 (34.5)	2 (11.8)	6 (42.9)	12 (44.4)	
No therapy	13 (22.4)	3 (17.6)	4 (28.6)	6 (22.2)	
Current imid-based therapy at TP5, *n* (%)	38 (65.5)	11 (64.7)	9 (64.3)	18 (66.7)	0.985
Current PI-based therapy at TP6 *n* (%)	9 (15.5)	5 (29.4)	1 (7.1)	3 (11.1)	0.161
Current antiCD38-based therapy at TP6, *n* (%)	17 (29.3)	6 (35.3)	4 (28.6)	7 (25.9)	0.8
Basis immunization, *n* (%)					0.564
COMIRNATY	53 (91.4)	16 (94.1)	13 (92.9)	24 (88.9)	
VAXZEVRIA	3 (5.2)	0 (0.0)	1 (7.1)	2 (7.4)	
VAXZEVRIA + COMIRNATY	1 (1.7)	1 (5.9)	0 (0.0)	0 (0.0)	
VAXZEVRIA + SPIKEVAX	1 (1.7)	0 (0.0)	0 (0.0)	1 (3.7)	
Boost immunization, *n* (%)					0.426
COMIRNATY	54 (93.1)	16 (94.1)	14 (100.0)	24 (88.9)	
SPIKEVAX	5 (6.9)	1 (5.9)	0 (0.0)	3 (11.1)	

This table includes all relevant disease- and patient characteristics of all patients within the long-term study cohort (present residual material for further analysis) stratified for BTI status (no infection, pre 3rd vaccination BTI and post 3rd vaccination BTI).

*CR* complete remission, *HDCT* high-dose chemotherapy, *IQR* inter-quartile range, *ISS* international severity score, *LC* light chain, *MM* multiple myeloma, *NA* not annotated, *PD* progressive disease, *PI* proteasome inhibitor, *PR* partial remission, *SD* stable disease, *VGPR* very good partial remission.

### Serological response assessment

The analysis of SARS-CoV-2 Spike IgG levels and in-vitro neutralization capacity against SARS-CoV-2 variants Delta and Omicron were performed at all timepoints as described previously [9, 10].

### T-cell response analysis

The SARS-CoV-2 specific T-cell response was assessed as reported previously, except for CD14 and CD20 antibodies, which were not used [9, 10, 21]. Briefly, IFN-y and TNF-a secreting T cells upon SARS-CoV-2 VOC-specific peptide stimulation (Table S7) were quantified in thawed PBMCs by flow cytometry.

### Analysis of CD4^+^ cytotoxic T-cells by flow cytometry

Cytotoxic T cells were quantified in thawed PBMCs of patients by flow cytometry. Surface and intracellular staining was performed according to the standard protocol (Supplementary material).

### Analysis of TNF-α stimulated NK-cells

After TNF-α treatment of PBMCs (Supplementary material), NK-cell populations were analyzed by flow cytometry upon surface and intracellular staining (Supplementary material).

### Single-cell CITEseq and data analysis

Thawed cells were washed and 1x10E6 cells were stained per donor for subsequent fluorescent-labeled sorting into T-, NK-, NKT- and B-cells (see Supplementary material). Sorted cells were incubated with master mix of each of 49 BD AbSeq oligonucleotide-conjugated antibodies (BD Biosciences; see Table S7). Single-cell capture and library preparation were performed according to the manufacturer’s instruction with the BD Rhapsody system (BD Biosciences and respective kits, see Supplementary material). Sequencing was run on a NextSeq2000 sequencer (Illumina). Raw data processing, quality control, normalization, multi-omics-factor-analysis-(MOFA)-based dimension reduction and clustering via graph-based Leiden algorithm were performed as outlined in the Supplementary material [22, 23]. Cell types were annotated using identified markers in the transcriptomic and AbSeq data (see Supplementary material). Differential abundancy testing between responders and non-responders in the cell populations was performed with the miloR package with providing “MM vs. HC”, timepoint and batch information as covariates (see Supplementary material) [24]. Differential expression analysis was performed on pseudobulk-aggregated data (see Supplementary material). Detailed (single sample) enrichment analysis was performed as described in the Supplementary material. To model the NK-cell subset along the differentiation pattern from CD56^bright^/CD16^dim^ to CD56^dim^/CD16^bright^, pseudotime analysis was performed using the monocle package (see Supplementary material) [25].

### Quantification and statistical analysis

For statistical analysis, R version 4.2.0 (local machine) and 4.2.2 (computation cluster) (The R Foundation for Statistical Computing) was used [26]. If not otherwise outlined, continuous variables were compared with the Mann-Whitney-*U* test for two independent groups and Kruskal-Wallis test for three or more independent groups, categorical variables with the Fisher’s exact test and the chi-square test.

## Results

### Immune cell phenotypes in patients with MM differ between responders and non-responders following SARS-CoV-2 vaccination

**Fig. 1:**
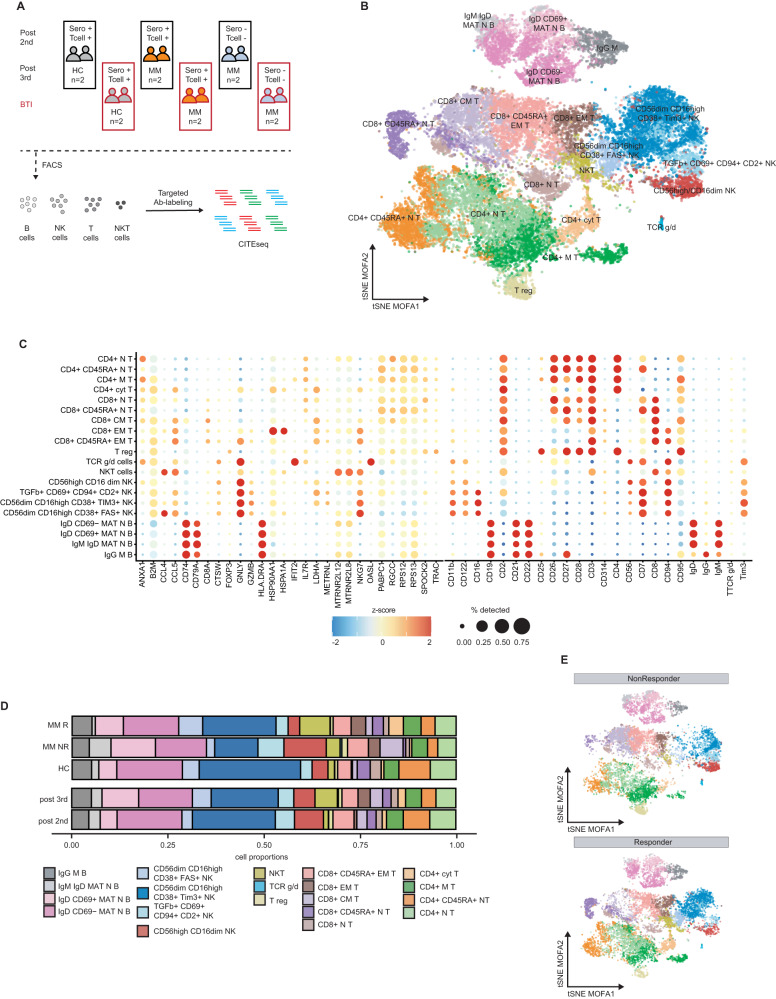
Peripheral immune cell phenotypes determined by scCITEseq in patients with MM undergoing SARS-CoV-2 vaccination and breakthrough infection. **A** Schematic representation of scCITEseq sample selection including donor vaccination response status determined after the 3rd vaccination, HC vs. patients with MM and the SARS-CoV-2 infection status, cell-sorting and scCITEseq approach (see also Supplementary material). **B** T-distributed stochastic neighbour embedding (t-SNE) visualization of integrated MOFA-factor dimensions derived by scCITEseq from 31,005 cells of 24 PBMC samples. **C** Dot plot visualization of the top 2 transcriptomic (left side) and surface proteome (right side) marker expression for each inferred cell type. **D** Bar plot of the proportion of inferred cell types normalized for number of sorted cells per donor for either timepoint post 2nd or post 3rd vaccination (bottom part) or for HC versus responders or non-responders (R/NR) among patients with MM (top part). **E** t-SNE visualization of integrated MOFA-factor dimensions for each measured cell for R/NR (exclusion of breakthrough infection). CM central memory, cyt cytotoxic, EM effector memory, HC healthy controls, M memory, MAT mature, N naive, NR non-responder, R responder, reg regulatory, Sero serological.

We previously observed improved serological responses after three doses of BNT162b2 against the SARS-CoV-2 wild-type (WT) strain in patients with MM (*n* = 100) [10]. Most patients with MM generated T-cell responses against the WT strain. However, serological and T-cell responses against other variants-of-concern were impaired [10]. Notably, insufficient humoral and/or T-cell response could partially be attributed to active anti-MM therapy or low CD19^+^ B-cell counts. To further investigate the differences between responders, non-responders and patients experiencing BTI, we performed single-cell cellular indexing of transcripts and epitopes sequencing (scCITEseq) from PBMC immune cell populations. We selected 11 representative individuals with controlling for disease- or treatment-related bias (Fig. S1A) from our previously published cohort for in-depth scCITEseq analysis resulting in 24 samples (Fig. 1A, see Supplementary material*)*. Samples were obtained from serological and T-cell (non-) responders after the 2nd (TP3) and 3rd (TP5) vaccination (Figs. 1A and S1A, B) [10]. We also included samples from 2 healthy controls (HC) after their respective 2nd and 3rd vaccination. As 35.2% of patients with MM (*n* = 37) from our main observation cohort (*n* = 105, Table S1) exhibited a BTI after the 3rd vaccination at the timepoint of data cut-off, we additionally included 2 full responders and 2 full non-responders among MM patients, along with 2 HC which all developed BTI after the 3rd vaccination. Samples after the 3rd vaccination and BTI were analysed via scCITEseq analysis (Figs. 1A and S1B).

After fluorescence-activated cell sorting (FACS, Fig. S1C) of the T-, NK-, NKT- and B-cells, the cells were labelled with 49 oligo-tagged surface markers relevant in characterizing immune cell compartments. After robust quality control of the sequencing data (Fig. S1D), we obtained data of 31,005 cells from 24 samples. Multi-omics factor analysis (MOFA) was used to integrate the surface proteome and whole-transcriptome expression data [22]. Latent factors associated to the sequencing batch were not included in the downstream analysis. Twenty distinct clusters were identified using Leiden clustering of cells in the latent space (Figs. S1E and 1B, C). In addition to the larger subpopulations among the CD8^+^ T-, CD4^+^ T-, NK- and B-cells, we identified small subpopulations such as TCR γ/δ and NKT-cells [27]. Further, this integrational analysis enabled the identification of activated naive CD8^+^ and CD4^+^ T-cells as well as CD8^+^ effector memory T- cells strongly positive to CD45RA (Fig. 1B, C).

While comparison of the immune cell composition after the second and third vaccination did not reveal any relevant differences (Fig. 1D), the comparison of MM non-responders to responders or HCs demonstrated varying fractions in the T- and NK-cell compartment (Fig. 1D, E). No disparities for B-cell sub-proportions were visible, but increased proportions of cytotoxic CD4^+^ T-cells were observed among MM responders as well as enumerated NKT-cells and increased CD56^dim^CD16^high^CD38^+^ NK-cells, hereinafter called mature CD38^+^ NK-cells (Fig. 1D, E).

Together, scCITEseq analyses of T-, B- and NK-cells after SARS-CoV-2 vaccination revealed relevant differences in T- and NK-cell subpopulations in MM vaccine-responders.

### Overrepresentation of cytokine responsive T-cell populations in patients with MM with a sufficient response to SARS-CoV-2 vaccination

**Fig. 2:**
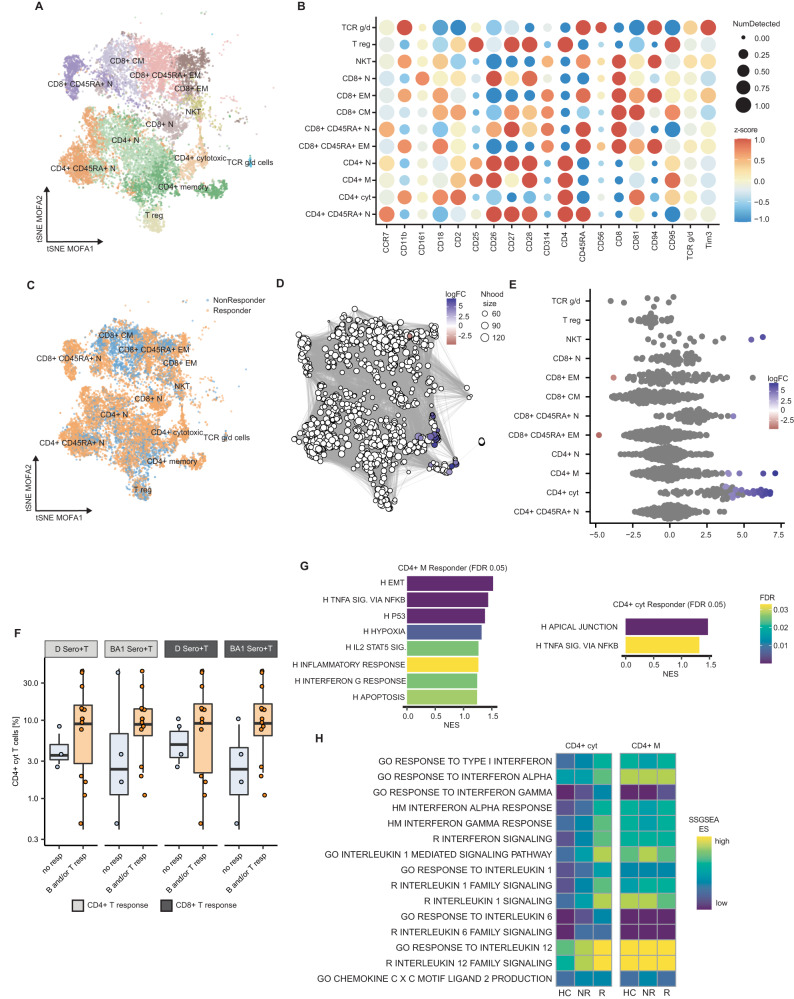
Cytokine-responsive T-cell populations in MM vaccination responders. **A** t-SNE visualization of integrated MOFA-factor dimensions derived from scCITEseq from 13,817 T-cells, colored by annotated cell type. **B** Top surface proteome markers per T-cell subpopulation. **C** t-SNE as in **A**, colored for response status after 3rd vaccination. **D** Visualization of inferred single-cell neighbourhoods and connections from milo framework (see methods and Supplementary material). Each neighbourhood colored regarding significant (spatial FDR < 0.2) differences in cells from R/NR. The size denotes the number of single cells grouped in the respective neighbourhood. **E** Cell type-based grouping of neighbourhoods, coloring regarding significant overrepresentation of R versus NR status per neighbourhood. **F** Frequency of CD4^+^ cytotoxic T-cells stratified for NR and serological and/or T-cell-R, splitted into CD4^+^ (light grey) and CD8^+^ T-cell-response (dark grey). CD4^+^-D-no resp *n* = 3, CD4^+^-D-B and/or T-resp *n* = 14; CD4^+^-BA1-no resp *n* = 4, CD4^+^-D-B and/or T-resp *n* = 13; CD8^+^-D-no resp *n* = 4, CD8^+^-D-B and/or T-resp *n* = 13; CD8^+^-BA1-no resp *n* = 4, CD8^+^-D-B and/or T-resp *n* = 13. **G** GSEA of results from differential expression analysis in respective cell types regarding R/NR-status. **H** Single-sample GSEA (SSGSEA) per donor-wise merged cell types of overrepresented subsets stratified for R/NR. Cytokine-responsive gene-sets from GO, HM and Reactome database. BA1 Omicron-BA.1-variant, CM central memory, cyt cytotoxic, D Delta-variant, EM effector memory, ES enrichment score, FDR false discovery rate, GO gene ontology, HC healthy controls, HM Hallmark, M memory, MAT mature, MM Multiple Myeloma, N naive, NES normalized enrichment score, Nhood neighbourhood, NR non-responder, R responder, reg regulatory.

Next, we studied T-cell compartment differences between vaccine-responders and non-responders. Twelve individual clusters were annotated based on their marker gene and surface protein expression (Figs. 2A, B and S2A). They resembled established T-cell populations covering naive CD4^+^ and CD8^+^ T-cells, CD8^+^ effector, effector memory and central memory T-cells, and also comprised small populations such as regulatory T-, TCR γ/δ and NKT-cells (Fig. 2B) [27]. Differential abundancy analysis with k-nearest neighbour (KNN) graphs was used to robustly test for differences in the single-cell populations, and each inferred neighbourhood was tested for representation of responder- versus non-responder-stemming T-cells while accounting for the timepoint after vaccination and MM vs. HC status and application of spatial false discovery rate (FDR) correction [24]. A significant overrepresentation of vaccine-responder-associated T-cells was observed in the CD4^+^ cytotoxic T-cell and to a lesser extent in the CD4^+^ memory compartment (Fig. 2C–E). High levels of these populations in the serological and/or cellular responders were validated using flow cytometry-based analysis of the CD4^+^ cytotoxic T-cell fraction, defined by co-expression of CD3, CD4, perforin, and/or granzyme (Fig. S2B), in an independent set of PBMCs derived from patients with MM after the 3rd vaccination (Fig. 2F). Sub-classification according to either B- or T-cell response did not reveal significant data owing to inter-individual variation in the immune responses; nonetheless trends to elevated levels among responders were observed when stratifying for WT, Delta or Omicron-related responses (Figs. 2F and S2C). When marker genes were investigated for the neighbourhoods with responder T-cell overrepresentation, these were associated with gene-sets related to interferon gamma (IFN-γ) and tumor necrosis factor alpha (TNF-α) signaling (Fig. S2D). Gene-set-enrichment-analysis (GSEA) of differentially expressed genes (responder vs. non-responder) in those cell types revealed a significant enrichment for similar terms associated with TNF-α and IFN-γ signaling (Fig. 2G). Additionally, single-sample GSEA (SSGSEA) on donor-wise merged pseudobulk data highlighted higher SSGSEA scores of gene-sets associated with Interferon- α (IFN-α), Interleukin 1 (IL-1) and 12 (IL-12) signaling in vaccine-responders compared to non-responders (Fig. 2H).

Together, these results highlighted significant variations in the T-cell compartment between vaccine-responders and non-responders, demonstrated the association between subsets of cytotoxic CD4^+^ T-cells with an improved B- or T-cell response, and IFN-α and TNF-α response/signaling to be pivotal in the altered T-cell populations following SARS-CoV-2 vaccination.

### Mature and cytokine-responsive NK-cell populations are associated with response to SARS-CoV-2 vaccination in patients with MM

**Fig. 3:**
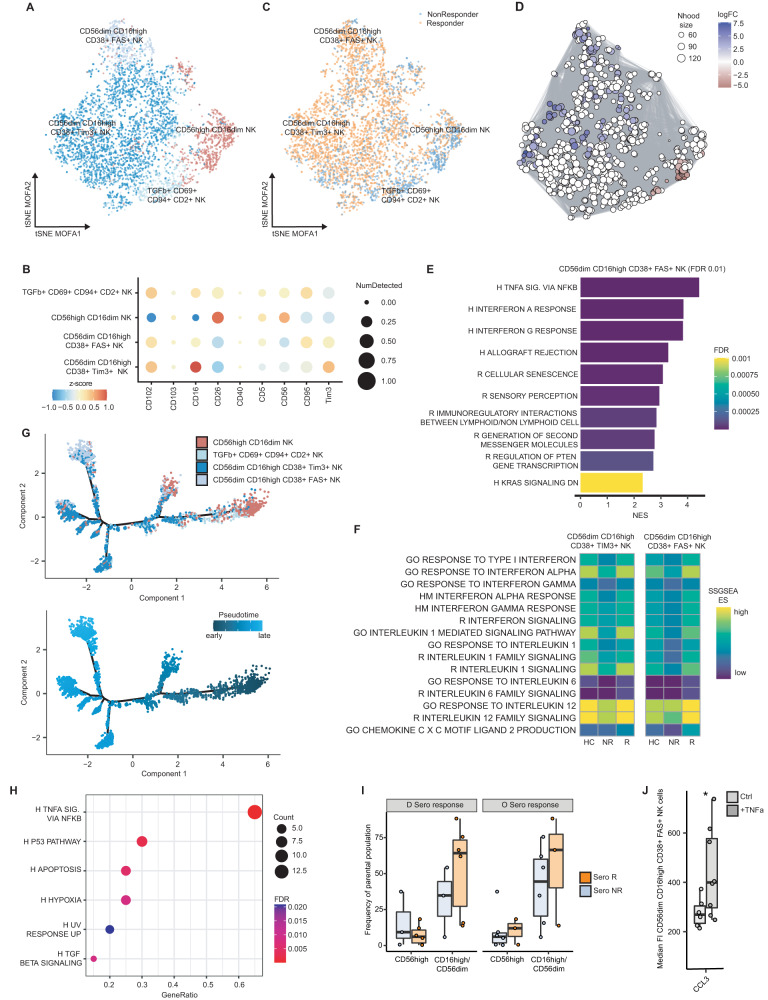
Mature and cytokine-responsive NK-cell populations associate with SARS-CoV-2 vaccination responders among patients with MM. **A** t-SNE visualization of integrated MOFA-factor dimensions derived from scCITEseq from 4,694 NK-cells, colored by annotated cell type. **B** Top surface protein markers for each NK-subset. **C** t-SNE as in **A**, colored for response status after 3rd vaccination. **D** Single-cell neighbourhoods and connections from milo framework (see methods and Supplementary material); neighbourhoods colored regarding significant differences (spatial FDR < 0.2) in cells from R/NR; size denotes number of single-cells per respective neighbourhood. **E** GSEA of results from differential expression analysis in respective NK-subset between R/NR. Hallmark and Reactome gene-set collection was used for GSEA. **F** Single-sample GSEA per donor-wise merged NK-subsets in R/NR. **G** Single-cell trajectories inferred by pseudotime analysis of single-cell NK-transcriptomes colored by pseudotime metric (early to late) or by respective NK-subset. **H** Hypergeometric overrepresentation analysis of differentially expressed genes alongside main trajectory using Hallmark gene-set database. **I** Frequency of NK-subsets stratified for serological R/NR, further divided regarding Delta- and Omicron-response. CD56^high^-D-no resp *n* = 3; CD56^high^-D-resp *n* = 6; CD16^high^/CD56^dim^-D-no resp *n* = 3; CD16^high^/CD56^dim^-D-resp *n* = 6; CD56^high^-O-no resp *n* = 6; CD56^high^-O-resp *n* = 3; CD16^high^/CD56^dim^-O-no resp *n* = 6; CD16^high^/CD56^dim^-O-resp *n* = 3. **J** MFI of CD16^high^/CD56^dim^CD38^+^FAS^+^-NK-cells stratified for control or TNF-α−treatment. Two-sided *t*-test, *p < 0.05. Ctrl control, ES enrichment score, HC healthy controls, NES normalized enrichment score, Nhood neighbourhood, NR non-responder, R responder.

Peripheral NK-cells are vital for the antiviral activity of the innate cellular immune response—especially in the context of COVID-19 [28–30]. Hence, NK-cells from all timepoints but without BTI were further investigated. Based on the surface marker profiles of the NK-cell clusters with regard to maturation and activation, we observed that one subpopulation exhibited high CD56 positivity. Most NK-cells were grouped into two mature CD56^dim^CD16^high^CD38^+^ NK-cell subsets, either expressing TIM3 or FAS (Fig. 3A, B). Further characterization of the NK-cell subpopulations revealed increased gene expression of cytotoxic *GZMA*, activation-associated *KLRF1*, cytokine and chemokine markers *IFNG*, *CCL3*, *CCL4*, *CCL5* and adhesion molecules *ITGB2* and *ITGAL* in the mature CD38^+^FAS^+^ subpopulation (Fig. S3A) [31]. The mature CD38^+^TIM3^+^ fraction of NK-cells exhibited high expression of cytotoxic *PRF1* and the cytokine/chemokine receptor *CXCR4*.

Next, all identified NK-cells were embedded in a KNN-graph, grouped into neighbourhoods which then were tested for overrepresentation of either vaccine-responders- or non-responders-associated NK-cells (Fig. 3C, D). Here, a substantial overrepresentation of mature CD38^+^FAS^+^ and to a lesser extent mature CD38^+^TIM3^+^ NK-cells was observed among vaccine-responders (Fig. 3D). GSEA of differentially expressed genes within the mature CD38^+^FAS^+^ NK-cells between vaccine-responders and non-responders revealed a strong enrichment pattern in TNF-α-signaling and IFN-α and -γ response-related gene sets (Fig. 3E). Further, SSGSEA displayed increased scores for IFN-α, IL-1 and IL-12 response/signaling gene sets in vaccine-responders compared to non-responders (Fig. 3F).

The identified peripheral NK-cell clusters differed in their grade of maturation and activation. Notably, NK-cells were ordered based on the inferred pseudotime trajectory with its main branch passing from the more immature CD56^high^CD16^dim^ NK-cells to the mature CD56^dim^CD16^high^CD38^+^FAS^+^ NK-cells (Fig. 3G). Because the mature NK-cells were highly overrepresented and the immature NK-cells underrepresented among vaccine-responders, monocle’s branched expression analysis modelling (BEAM; see methods) was used to identify genes that differ from the early to the late state across the main branch of the trajectories (Fig. S3B) [25]. Here, we observed a characteristic enrichment of TNF-α signaling-related genes (Fig. 3H). Furthermore, we observed an increased frequency of mature NK-cells in serological responders for Delta- and Omicron-induced vaccination (Figs. 3I and S4A). However, we did not observe a difference in NK subpopulations when patients were stratified according to T-cell responses (Fig. S4B). Notably, the scCITEseq phenotypes regarding mature CD38^+^FAS^+^ NK-cells with CCL3 and CCL5 expression and TIM3^+^ with perforin expression were also confirmed via flow cytometry (Fig. S4C). We further performed functional validation using TNF-α in vitro stimulation mimicking the impact of cytokine-induced signaling in NK-cells. Strikingly, mature CD38^+^FAS^+^ NK-cells showed a relevant TNF-α responsiveness in terms of increased CCL3 surface expression (Figs. 3J and S4D).

In sum, these findings emphasize an increased fraction of mature CD38^+^ NK-cells, either FAS- or TIM3-expressing, in vaccine-responders among patients with MM, which show a cytokine responsive phenotype, particularly after in vitro TNF-α stimulation. Importantly, overrepresentation of these NK-cell populations was not exclusively to T-cell responders, further highlighting the independent role of innate immunity-related NK-cells.

### High pan-variant vaccination response levels after SARS-CoV-2 BTI with similar cytokine-responsive single-cell patterns in MM

**Fig. 4:**
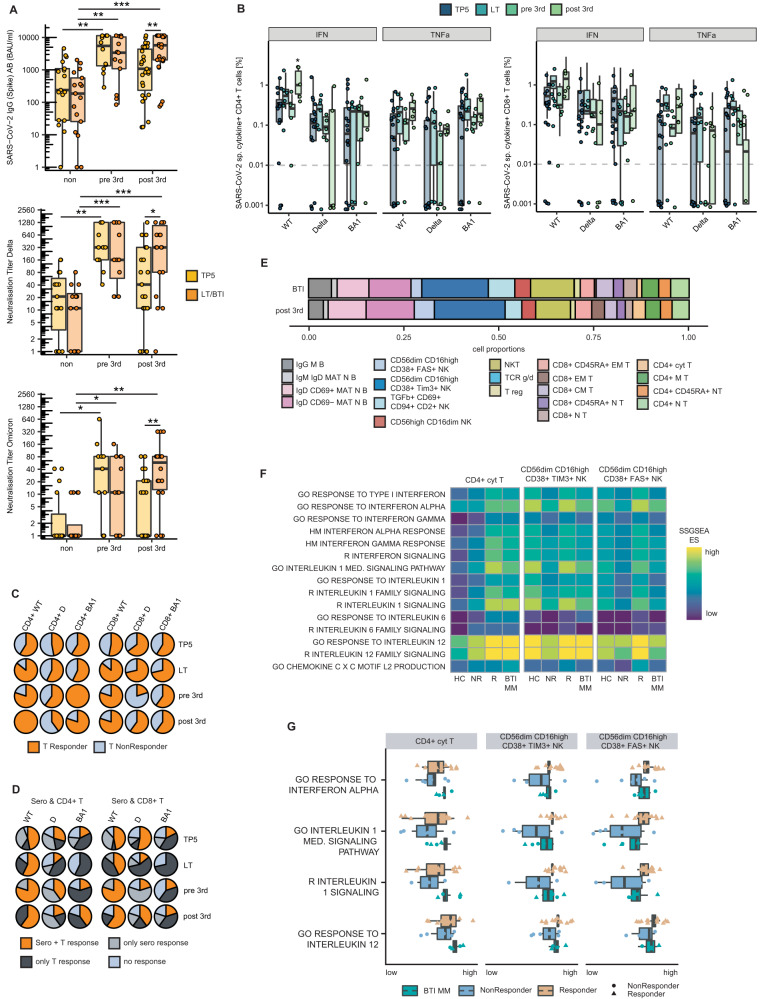
Vaccination response and single-cell enrichment patterns after SARS-CoV-2 breakthrough infection in patients with MM. **A** Serum anti-Spike IgG-levels and Delta- or Omicron-variant neutralization titers in MM patients differing in SARS-CoV-2-breakthrough infection (BTI)-status: without any (*non*), BTI before the 3rd vaccination (*pre 3rd*) or after (*post 3rd*) stratified for timepoints 21-28 days (light orange) and long-term 2-6 months after the 3rd vaccination (in case of *post 3rd* BTI, the long-term timepoint was 21-28 days after BTI). Spike IgG non-TP5 *n* = 17, non-LT/BTI *n* = 17; pre 3rd-TP5 *n* = 12, pre 3rd-LT/BTI *n* = 13, post 3rd TP5 *n* = 27, post 3rd-BTI *n* = 26; NT Delta-non-TP5 *n* = 15, non-LT/BTI *n* = 16; pre 3rd-TP5 *n* = 12, pre 3rd-LT/BTI *n* = 12, post 3rd-TP5 *n* = 25, post 3rd-BTI *n* = 18; NT-Omicron non-TP5 *n* = 15, non-LT/BTI *n* = 16; pre 3rd-TP5 *n* = 13, pre 3rd-LT/BTI *n* = 12, post 3rd-TP5 *n* = 25, post 3rd-BTI *n* = 18. **B** Frequency of CD4^+^-or CD8^+^-SARS-CoV-2-specific and cytokine + T-cells after stimulation with indicated variant-peptides. Data stratified for TP5, non-LT, pre 3rd-BTI LT and post 3rd-BTI (see **A**). For n/condition, see Table S2A, B. **C** Fraction of T-cell-R/NR indicated per variant and timepoint. T-cell response information stratified in CD4^+^ (left) or CD8^+^ (right). For n/condition, see Table S3. **D** Fraction of NR (blue), only serological-R (light grey), only T-cell-R (dark grey) and full (serological and T-cell) R (orange). T-cell-R/NR stratified in CD4^+^ (left) or CD8^+^ (right), For n/condition, see Table S4. **E** Proportion of cell types by scCITEseq stratified for post 3rd or after BTI. **F** SSGSEA per donor-wise merged cell types in R/NR (see Fig. 2C–E or Fig. 3C, D). Cytokine-responsive gene-sets from GO, HM and Reactome database. **G** SSGSEA enrichment scores for R (light orange, *n* = 12 [MM *n* = 8, HC *n* = 4]) or NR (blue, all MM *n* = 6) after 3rd vaccination among MM, contrasted to SSGSEA ES from MM after BTI (green, all MM *n* = 4). Circles denote NR-status and triangles R-status. BAU binding antibody units, BA1 Omicron variant BA.1, cyt cytotoxic, D Delta variant, ES enrichment score, IFN interferon-γ, IMM infected patients with MM, LT long term, M memory, MM Multiple Myeloma, NR non-responder, R responder. Statistical testing with Wilcoxon-test, *P*-values as * < 0.05; ** < 0.01 and *** <0.001.

Next, we investigated whether BTI influences the immune response and monitored our observation cohort (*n* = 105, Table S1) for occurrence of BTI for up to 6 months. At the timepoint of data cut-off, material of 58 patients (present study cohort) was present for further immune-profiling analysis. Here, 24.1% (*n* = 14) exhibited a SARS-CoV-2 infection any time before completing the three-vaccination-course (labelled as *pre 3rd*), 46.6% (*n* = 27) were infected with SARS-CoV-2 after the 3rd vaccination (labelled as *post 3rd*) and 29.3% (*n* = 17) never experienced a BTI (labelled as *non*) (Table 1). No differences regarding MM-associated clinical baseline characteristics were observed after the 3rd vaccination (TP5) and the long-term timepoint or BTI (LT/BTI) evaluation (Table 1). Interestingly, most patients who did not experience a BTI were under active MM therapy at TP5 (*p* = 0.012). When serological response levels were evaluated, patients who experienced a BTI at any time before, during or after their vaccination course showed remarkably higher SARS-CoV-2-IgG levels and neutralization titers (NT) against Delta and Omicron compared to those never infected (Fig. 4A). A significant increase from TP5 to LT/BTI was only observed in patients with an infection after the 3rd vaccination (Fig. 4A). Waning of serological responses was not observed in those patients infected before receiving the 3rd vaccination (Fig. 4A). With the aim to further evaluate the T-cell response at TP5 and LT/BTI, frequencies of CD4^+^ or CD8^+^ SARS-CoV-2-specific cytokine-positive T-cells after stimulation with WT, Delta or Omicron BA.1 peptides were measured (see methods and Fig. S5A, B). Typically, SARS-CoV-2-specific T-cell frequencies were lower after stimulation with Delta and BA.1 than with WT (Fig. 4B). Post 3rd infected patients exhibited higher levels of CD4^+^ SARS-CoV-2 WT IFN-γ positive T-cells at BTI than TP5 (Fig. 4B). To compare fractions of T-cell responders at the different timepoints and across variants, patients were considered responders if they exhibited measurable SARS-CoV-2 specific T-cells (≥0.1%) for both cytokines after the respective variant-peptide stimulation (Figs. 4B and S5A, B). Increased fractions were observed for the CD4^+^ and CD8^+^ T-cell responses against WT in post 3rd infected patients compared to TP5 (Fig. 4C). Such trends were also observed for BA.1. Most patients showed a serological and/or T-cell response against the WT strain after the 3rd vaccination and the rate of both-level responders for the WT strain increased in post 3rd infected patients after the infection (Fig. 4D). The response fractions were more heterogeneous for the Delta and Omicron variant with a similar trend towards higher serological and/or CD4^+^ T-cell response for BA.1 (Fig. 4D).

To study the effect of BTI at single-cell resolution, two serological/T-cell responders and serological/T-cell non-responder among the patients with MM as well as two HC who were infected with SARS-CoV-2 after the third vaccination were included in the scCITEseq analysis (Figs. 1A and S1B). The already processed single-cell dataset with the generated clustering and cell type assignment was therefore investigated for alterations between the third vaccination and BTI. No differences in compartment compositions and in graph-based differential abundance testing were observed (Figs. 4E and S5C, D). However, comparing the single-sample enrichment patterns in those cell types overrepresented among vaccine-responders at TP5, SSGSEA revealed high scores for cytokine-responsive gene sets in MM with BTI similar to MM responders after the 3rd vaccination but higher in contrast to vaccine-non-responders (Fig. 4F). In this context, the elevated SSGSEA scores in the cytokine-responsive gene sets were observed in the post 3rd infected patients with MM regardless of their previous vaccination responder status. (Fig. 4G).

Collectively, these data highlight elevated serological and/or T-cell response levels in patients with MM who experienced BTI at any timepoint during their vaccination course. Importantly, the highest levels were observed for the WT strain pointing towards a generally sufficient immunogenicity in patients with MM but more heterogenous immune responses against other variants. At BTI after 3 doses of SARS-CoV-2 vaccine, the same cellular cytokine-responsive enrichment patterns were observed as identified in vaccine-responders, regardless of the prior vaccination response status.

## Discussion

To enable a detailed characterization of peripheral immune cells in context of SARS-CoV-2 vaccination and BTI, we applied multi-modal scCITEseq on 24 samples from 11 individuals (7 patients with MM [all in at least VGPR and first line of treatment to limit bias] and 4 HC) spanning from the timepoint after 2nd and 3rd vaccination and BTI. The experimental set up used in this study allowed for 1) detailed characterization of selected peripheral immune cells at single-cell resolution and at different timepoints during the course of vaccination 2) investigation of immune cell types associated with different response statuses and 3) exploration of serological and functional T-cell responses as well as single immune cell patterns in context of BTI.

To investigate beyond the humoral and functional T-cell response, we enriched the PBMCs obtained after the 2nd and 3rd vaccination and BTI for B-, NK- and T-cells for subsequent scCITEseq [9, 10]. These broad populations were selected owing to their direct association with the B-cell mediated humoral or functional T-cell responses, and their relevance to innate immunity-related response to viral infections, particularly severe COVID-19 [28–30, 32–34]. Here, multi-modal scCITEseq generated an in-detail and well-interpretable peripheral immune cell dataset of key cell types in antiviral vaccination in patients with MM.

In vaccination responders, a significant overrepresentation of CD4^+^ cytotoxic T-cells and to a lesser extent CD4^+^ memory T-cells was observed. CD4^+^ cytotoxic T-cells represent a scarce T-cell population that is characterized by expression of cytotoxic gene patterns usually associated with CD8^+^ cytotoxic T-cells and can be identified in single-cell sequencing studies. Here, they were characterized by a prominent CD4^+^ T-cell surface marker profile and characteristic transcriptomic expression pattern including *NKG7*, *GNLY*, *GSMB*, *PRF1*, *CCL4* and *CCL5* [27, 35]. Notably, CD4^+^ cytotoxic T-cells are essential for protective immune responses to viral infections and vaccines against pathogens such as Influenza A and others, and showed a strong enrichment in patients with COVID-19 [36–40]. Furthermore, in CD4^+^ memory T-cells, a subcluster with similar cytotoxic gene expression, was observed and hypothesised as CD4^+^ cytotoxic precursor T-cells [35]. This might explain the limited but present overrepresentation of CD4^+^ memory T-cells—possibly resembling this CD4^+^ cytotoxic precursor phenotype—in vaccine-responders. We further observed trends towards higher CD4^+^ cytotoxic T-cells in humoral and/or T-cell responders in MM after the 3rd vaccination. Enrichment analysis showed a strong signal toward increased TNF-α and INF-γ signaling as well as INF-α, IL-1 and IL-12 response/signaling. In a single-cell study of T-cell phenotypes in supercentenarians linking high fractions of CD4^+^ cytotoxic T-cells to repeated viral exposure and favourable anti-tumor immunity, extracted CD4^+^ cytotoxic T-cells secreted TNF-α and IFN-γ upon ex vivo stimulation [41]. Additionally, CD4^+^ cytotoxic T-cell associated IFN-γ production was linked to protection against malaria [42]. Hence, increased levels of CD4^+^ cytotoxic T-cells are a relevant hallmark of adequate immunization.

Similar to that of CD4^+^ cytotoxic T-cells, we identified a significant overrepresentation of CD56^dim^CD16^high^CD38^+^FAS^+^ NK-cells and to a lesser extent CD56^dim^CD16^high^CD38^+^TIM3^+^ NK-cells in vaccine-responders after the 3rd vaccination. In general, NK-cells participate in the innate immunity against pathogens, particularly viruses, releasing cytotoxic granules or CD16-mediated antibody-dependent cellular toxicity [28]. Upon viral infection, CD56^dim^CD16^high^ NK-cells are able to convey immunity either in cytokine- or receptor-driven mechanisms [28]. NK-cell response following SARS-CoV-2 infection reflects a hallmark of early response with expression of CD38, *TIM3*, *TIGIT* and cytotoxic perforin and granzyme B [19, 28, 29]. Lung-recruited NK-cells showed a characteristic chemokine expression pattern featuring high *CCL3* and *CCL4* expression [43, 44]. NK-cells that were significantly overrepresented in vaccine-responders showed high CD16 and CD38 expression levels. A trend towards higher CD16 expression levels among serological vaccine-responders was observed in an independent set of PBMCs obtained after the 3rd vaccination of MM patients. Mature CD38^+^FAS^+^ NK-cells further showed elevated transcript levels of *GZMA, CCL3, CCL4* and *CCL5*, emphasizing their antiviral targeting and tissue migrating potential. Additionally, mature CD38^+^TIM3^+^ NK-cells displayed prominent *PRF1* and *GZMB* expression profiles. Vaccine-response-associated expression patterns in these NK-cell subtypes showed a strong enrichment of type-I-interferon, TNF-α- and IL-12-mediated signaling. Furthermore, we modelled the measured NK-cells along their differentiation pseudo-time trajectory, since NK-cell differentiation resembles a continuum rather than strict discriminative states [28]. The immature CD56^high^ NK-cells and the mature CD56^dim^CD16^high^CD38^+^/FAS^+^ NK-cells assorted at opposite ends of the trajectory. Differential genes along the trajectory resembled those associated with TNF-α− and NFκB-mediated signaling in line with NK responses after flavivirus- or Influenza A infection, where an increased rate of infections was observed if NK-cell activating antibodies were absent after vaccination [45–47]. Upon in vitro stimulation with TNF-α, we observed significantly higher CCL3 levels in mature CD38^+^FAS^+^ NK-cells. Together, these results emphasize representation of highly mature NK-cell populations with an active antiviral phenotype, evidenced by the expression of individual markers and by a strong enrichment in relevant cytokine-responsive molecular programs.

Despite previous vaccinations, many patients with MM exhibit SARS-CoV-2 BTI, and previous studies suggested both relevant similarities or differences between vaccination- and infection-induced responses [16, 48–51]. Accordingly, in a sub-cohort of 58 patients with MM, we identified increased serological responses in patients with either pre 3rd or post 3rd BTI in comparison to non-infected patients with MM. BTI after the 3rd vaccination resulted in a significant boost in serological response levels. Generally, in SARS-CoV-2, the changing variant spectrum also affects the immune responses after vaccination and infection. In this context, a variant-dependent decrease was observed, with adequate serological WT but impaired Omicron NT, in line with previous studies determining a dependence of higher serological titers on heterogeneous immunization events (vaccination plus infection) and the respective variants [49, 51]. Regarding functional T-cell immunity, we observed that CD4^+^ and CD8^+^ T-cell levels were generally higher for WT-associated responses compared to Delta or Omicron. Accordingly, significantly higher levels of SARS-CoV-2 WT-specific CD4^+^ T-cells were observed for the comparison of post 3rd infected individuals versus TP5 (directly after 3rd vaccination). Despite individual trends towards higher fractions of T-cell responders at the post 3rd BTI timepoint, no significant differences were detected. This aligns with previously published data where no major differences for SARS-CoV-2 specific CD4^+^ or CD8^+^ T-cells were observed in different immunization sequences spanning from 3-course vaccination without infection to different timepoints/virus-variants plus vaccination combinations [49]. The highest fraction of serological and/or T-cell vaccine-responders at TP5 and a relevant increase by additional infection-based immunization was observed for the WT variant. This highlights the general immunogenicity but also variant dependency in patients with MM. To address the differences at single-cell resolution and to study similarities between vaccination- and infection-induced responses, we performed scCITEseq on sorted PBMCs collected after post 3rd vaccination BTI. While cell type proportions at TP5 and after BTI suggested certain differences in the T-cell and NK-cell compartment, such differences were not statistically evident. This aligns with the previous data suggesting immunization-induced cellular programs being present in both events and differences of vaccination to natural infection being mostly present in clonality dynamics [48, 52]. Importantly, the cytokine-responsive enrichment patterns observed in overrepresented cell types among vaccine-responders were also recovered in these cell types after BTI regardless of the previous vaccination response status. This cytokine responsiveness may highlight the link between immunization responses to mRNA vaccination and natural infection. BNT162b2 mRNA vaccine-mediated responses included a systematic signature of increased IFN-y, TNF-α, and CXCL10 expression, as well as a coordinated release of IL-1Ra and CCL4 to positively correlate with anti-Spike-RBD antibodies [53]. These results were noted upon booster and single vaccination of SARS-CoV-2 convalescent individuals and agreed with the cytokine-responsive phenotype observed among responders/BTI. Thus, eliciting strong cytokine expression after mRNA vaccination with addition of immunostimulants or adapted delivery systems might support increased immunological responses, which extends from anti-viral to anti-tumor vaccination, particularly in immunocompromised patients [54].

Overall, we investigated peripheral immune cell compartments in context of immunological responses after SARS-CoV-2 vaccination and BTI, demonstrating elevated but variant-dependent serological and T-cell responses. Remarkably, single-cell resolution and functional validation assays revealed that cytokine-responsive CD4^+^ cytotoxic T-cells and CD56^dim^CD16^high^CD38^+^ either FAS^+^/TIM3^+^ NK-cells were highly associated with response to vaccination and BTI. These results expand our understanding of molecular immune cell patterns associated with immunization responses and may lead to an improved design of vaccination strategies.

### Supplementary information

Supplemental Material

## Data Availability

The single-cell CITE sequencing data in format of raw counts have been deposited at GSE229187 and are publicly available as of the date of publication. Any additional information required to re-analyze the data reported in this paper is available from the lead contact upon request.

## Code availability

The analysis code involved in the single-cell CITE sequencing data analysis is deposited in the following GitHub repository: https://github.com/jenssle/CITEseq_MM_CoV_Vac. Any additional information required to re-analyze the data reported in this paper is available from the lead contact upon request.
