# Supplementary material for: Cytokine-responsive T- and NK-cells portray SARS-CoV-2 vaccine-responders and infection in multiple myeloma patients
Source: Leukemia. 2023 Dec 4;38(1):168–80. doi: 10.1038/s41375-023-02070-0 (PMC10776400; doi:10.1038/s41375-023-02070-0)
Supplement: Supplementary file 1 — Supplemental Material [file 41375_2023_2070_MOESM1_ESM.pdf]

Supplementary Materials

Cytokine-responsive T- and NK-cells portray SARS-CoV-2 vaccine-responders and infection in multiple myeloma patients

Julius C. Enssle, Julia Campe, Alina Moter, Isabel Voit, Alec Gessner, Weijia Yu, Sebastian Wolf, Björn Steffen, Hubert Serve, Melanie Bremm, Sabine Huenecke, Michael Lohoff, Maria Vehreschild, Holger F. Rabenau, Marek Widera, Sandra Ciesek, Thomas Oellerich, Katharina Imkeller, Michael A. Rieger, Ivana von Metzler & Evelyn Ullrich

Supplemental methods

Figs. S1 to S5

Tables S1 to S7

SUPPLEMENTAL METHODS

Sample and clinical data acquisition

All individuals received the vaccination courses highlighted above. The interval from first to second vaccination was 21-42 days and the timespan between second and third vaccination was 3-6 months. In cases of a 2nd booster application, the timespan between the third and fourth vaccination was also 3-6 months. Biobanking of serum and was performed as described previously (see also **Fig.S1B**).[1] The different basis and boost vaccinations per group are depicted in Table S1 and S2 for the observation and the present study cohort. In the study cohort (n=58), 17 patients with MM received a 2nd booster (n=16 BNT162b2 and n=1 Spikevax), which were equally distributed between the long-term follow-up without BTI (n=8) and the BTI subgroups (n=9). All individuals (including healthy controls) who are part of the scCITEseq cohort (11 representative individuals, in sum 24 samples with one donor contributing samples once after 2nd vaccination, twice after third vaccination [measured in two different sequencing runs] and once after BTI. If necessary, the respective analyses were adjusted in this regard.) were selected according to their serological and T-cell response status (see Results section) as published previously and received three doses of BNT162b2 during the course of vaccination.[1,2] PBMCs were isolated from whole blood of patients and healthy controls and cryopreserved as reported previously.[1] 3-6 months after the last vaccination, an additional biobanking was performed to allow investigation of long-term or BTI follow-up. Patients were routinely seen at our institution and PCR-based testing for SARS-CoV-2 infection was performed in case of any clinical signs of an infection. Additionally, anti-nucleocapsid (NC) IgG antibody testing was performed before the first vaccination, 2-3 months after the second and every 2-3 months after the third vaccination. Relevant clinical and disease-related data were retrieved from the electronical health-care-record system at our institution.

Thawing of PBMCs

Cryopreserved PBMCs were thawed with a concentration of 1 to 1.5x10^6 cells/ml in RPMI1640 supplemented with 10% FBS (Gibco). Afterwards the cells were washed and rested for 1h in RPMI 1640 containing GlutaMAX (Gibco), 10% FBS and 1% penicillin/streptomycin (Gibco) at 37°C, 5% CO_2.

Surface and intracellular staining for flow cytometry

For detection of extra- and intracellular markers via flow cytometry thawed PBMCs were used. First, cells were blocked with human IgG antibody (Concentration: 0,1 g/l) and stained with Fixable Viability Stain 510 (BD Biosciences) for live/dead cell discrimination according to the manufacturer's instruction. Surface staining was performed in brilliant stain buffer by using fluorochrome-conjugated antibodies as specified in the according method sections. After fixation with 3.7% formaldehyde and permeabilization with Saponin buffer (PBS +0.2% Saponin [Sigma-Aldrich] + 1% bovine serum albumin [BSA, Sigma Life Science]), PBMCs were stained intracellular. For analysis of CD4+ cytotoxic T-cells, surface staining the fluorochrome-conjugated antibodies against CD3 (clone UCHT-1, Biolegend), CD4 (clone SK3, BD Biosciences), CD8 (clone SK1, BD Biosciences), CD56 (clone NCAM16.2, BD Biosciences), CD19 (clone SJ25C1, BD Biosciences) and CD14 (clone MϕP-9, BD Biosciences) and intracellular staining with anti-human granzyme B (GzmB, clone REA226, Miltenyi) and anti-human perforin (clone δG9, Biolegend) antibodies was performed. Cells were measured by BD FACSCelesta™ Flow Cytometer (BDBiosciences) and data were analyzed with FlowJo™ Software (v10.3, BD Biosciences).

TNF-α treatment of PBMCs in vitro

Thawed PBMCs were seeded in a concentration of 1x10^6 cells/ml in RPMI 1640 supplemented with GlutaMAX, 10% FBS and 1% penicillin/streptomycin in a flat bottom 24-well

plate and treated with 10 ng/ml recombinant human TNF-α (Peprotech) for 24h. Control samples were treated with 0.001% BSA. For intracellular cytokine detection, 2µM BD GolgiStop™ (BD Biosciences) were added 4h prior to flow cytometry staining. For surface staining the following fluorochrome-conjugated antibodies were used: CD56 (clone MY31), CD16 (clone 3G8), CD18 (clone mAb24), CD38 (clone HB7), CD3 (clone UCHT-1), CD19 (clone SJ25C1), CD14 (clone MϕP-9) and CD95 (clone DX2) (all BD Biosciences). Intracellular staining was performed by using anti-human CCL3 (clone D21-1351, BD Biosciences). Additional intracellular staining was performed with the corresponding Isotype control (BD Biosciences) to assess signals by unspecific binding.

Flow cytometry cell sorting for scCITEseq analysis

As recommended, equal amounts (in µg) of fluorescent-labeled (FACS) and oligonucleotide-labeled (AbSeq) anti-CD3 and anti-CD56 antibodies were added to the washed cells to allow appropriate antibody staining for both read-outs. Both antibodies were from the same clone (SK7 for CD3 and NCAM16.2 for CD56). After 10 minutes of incubation on ice, the remaining fluorescent-labeled antibodies (anti-CD19, anti-CD14, anti-CD45, see **Tab.S7**) and sample tags were added and incubated for another 30minutes. Samples were washed and labeled with 7AAD for live/dead discrimination. Cells were sorted with the BD FACS Aria Fusion device (BD Biosciences) with a 70µm nozzle under sterile conditions. Per donor, equal amounts of T-cells (defined as 7AAD-, CD45+, CD19- CD14-, CD56-, CD3+; $3x10^4$ cells), B cells (7AAD-, CD45+, CD19+, CD14-; $1x10^4$ cells), NK-cells (defined as, 7AAD-, CD45+, CD19-, CD14-, CD56+ CD3-; $1x10^4$ cells) and NKT-cells (defined as 7AAD-, CD45+, CD19-, CD14-, CD56+ CD3+; $5x10^3$ cells) were aimed and sorted in the same tube to gain constant cell distributions between donors (see **Tab.S6** and **Tab.S7**, **Fig.S1C**). Sorting purity of the different populations

was verified by a test sort and reanalysis of the different populations prior to sorting. Purity of the desired populations from lymphocytes was >90%.

Single-cell capture and barcoding

After sorting, cells were washed once with 3 ml Stain Buffer (BD Biosciences, cat# 554657), and resuspended in 100 µl Stain Buffer. Then, a master mix containing 2 µl of each of 49 BD AbSeq oligonucleotide-conjugated antibodies (BD Biosciences; see **Tab.S7**) was prepared and added to the cell suspension before incubating for 40 min on ice. The cells were washed twice with 3 ml BD Stain Buffer and resuspended in 620 µl BD sample buffer (BD Biosciences, cartridge reagent kit cat# 633731). To assess the cellular concentration and viability, cells were first stained with 3.1 µl Calcein AM (2 mM, BD Biosciences, cat# 564061) and 3.1 µl Draq7 (BD Biosciences, cat# 564904) for 5 min at 37 °C in the dark, before 10 µl of the cell suspension in a disposable hemocytometer (Incyto, cat# DHC-N01-5) were analyzed with the BD Rhapsody scanner. A cell suspension containing 40000 captured cells was prepared and immediately loaded on the BD Rhapsody microwell cartridge (BD Biosciences, cat# 633733) according to manufacturer's instructions. Then, oligonucleotide-labeled beads were added to the cartridge before a washing step was performed and cells were lysed by adding lysis buffer (5 mM DTT) and incubating for 2 min at RT. To perform reverse transcription and exonuclease I treatment of the mRNA that is captured on the beads together with Sample Tags and oligonucleotide-labeled antibodies, beads were retrieved from the BD Rhapsody cartridge (BD Biosciences, cDNA kit, cat# 633773).

Library preparation and sequencing

Following the instructions of the BD Rhapsody whole transcriptome analysis (WTA), AbSeq and Sample Tag amplification kit (BD Biosciences, cat# 633801), DNA libraries were generated. The final libraries including indexes for Illumina sequencing were assessed regarding their concentration and size with a Qubit 4 Fluorometer using a High Sensitivity dsDNA kit

(ThermoFisher Scientific, cat# Q32851) and an Agilent 4150 TapeStation using a High Sensitivity D1000 ScreenTape (Agilent, cat# 5067-5584, 5067-5585). At last, library pools with a final concentration of 1 nM were mixed and loaded in multiple runs (75 bp paired-end) on a NextSeq2000 sequencer (Illumina) together with 20 % PhiX DNA.

Read alignment, quantification and multiplet identification

After Illumina sequencing, the SevenBridges platform (Seven Bridges Genomics, https://www.sevenbridges.com) was used to upload the FASTQ files and reference sequences for mRNAs and AbSeqs. The data was then processed using the BD Rhapsody analysis pipeline (BD Biosciences) with default settings. In summary, low quality read pairs were ejected before the leftover R1 reads were mapped for the identification of cell label and unique molecular identifier (UMI) sequences. Second, the R2 reads were annotated to the reference sequences allowing reads with identical cell label, UMI sequence and reference gene to be collapsed into a single molecule. With the help of a recursive substitution error correction (RSEC) algorithm, the molecule counts were corrected for sequencing errors. Then, Sample Tags were mapped to cells, cell counts were determined to create RSEC-adjusted molecule count matrices and multiplets were identified.

Quality control, normalization, embedding and clustering

For all subsequent data processing and analysis steps, the Orchestrating Single-cell Analysis (OSCA) framework was used.[3] After removal of multiplet- and invalid-labelled cells, the resulting single-cell experiment (sce) object was splitted for the data modalities whole-transcriptome (WTA) expression and AbSeq expression. Zero-count containing cells in the AbSeq data were removed to account for ambient-solution contamination. AbSeq data was processed via bimodal distribution (majority of non-upregulated and minority of upregulated protein targets) estimation, median based size-factor computation for the not-upregulated majority followed normalization and log-transformation utilizing the respective size factors.[3] Additionally, dead and

low-quality cells were removed by identifying outliers for upper/lower library size, low number of detected features and high percentage of mitochondrial transcripts in the WTA data. Again, size factors were estimated and scaling normalization and log-transformation was applied to the WTA data.

To embed the different data levels into a shared dimension reduction, multi-omics factor analysis (MOFA) was used with default options.[4] Since the respective data was acquired by two sequencing runs, the inferred MOFA latent factors (LF) were investigated for association with the known batches via logistic regression. Those factors strongly associating with the sequencing batch were removed and the remaining latent factors were used for clustering via graph-based Leiden algorithm.[5]

Cell type annotation

For annotation of cell types, marker features in the transcriptomic and AbSeq data were identified using scran's *scoreMarkers()* function for each identified cluster while blocking for the batch vector. On the basis of the inferred marker features, cells were grouped into the B-cell, T-cell (including NKT-cells) and NK-cell compartment. These subsets were then again subclustered following the above highlighted approach. For each sub-clustering, the MOFA LFs that associated with the batch vector were again removed. Marker features were again identified using *scoreMarkers()* and individual cell types were assigned in an expert-based fashion by the respective marker feature sets.[6,7]

Differential abundancy testing

To test for differences in cell type abundancies between responders and non-responders, the miloR package was utilized.[8] Therefore, the T- or NK-cell subsets were embedded in a k-nearest-neighbour graph on the basis of the previously inferred MOFA factors without batch-associating LFs. The embedded cells were further assigned to representative neighbourhoods and

cells per neighbourhood were counted. Since NKT-cells were separately sorted prior to single-cell capture, the NKT-cell counts after sorting were included into the design matrix for the subsequent testing (beside the experimental levels response status, MM vs. HC, timepoint and batch). After computation of the neighbourhood connectivity, the differences in abundancies were tested using the QL method in edgeR and the spatialFDR was used to control for multiple testing as highlighted by the authors.[8] Differences in abundancy per neighbourhood between responder status was considered relevant with a FDR of 0.2. These neighbourhoods were then subjected for further analysis.

Differential expression analysis

To account for p-value inflation in single-cell differential expression analysis, a merged single-cell object with aggregating gene counts over donor and cell type was created via *aggregateAcrossCells()* from the scuttle package using default settings. The resulting pseudobulk object was filtered for sufficient cell numbers per pseudobulk sample (≥5). Differential expression testing was performed via the *pseudoBulkDGE()* wrapper function between responder and non-responders per individual cell type with providing batch, timepoint and MM vs. Ctrl status as covariates in the experimental design matrix. Differentially expressed genes were considered significant with a FDR of 0.1.

Gene set enrichment analysis

In the cell types found to be significantly overrepresented in responders versus non-responders, differential expression analysis was performed as depicted above and genes were ranked by the F-statistic. The resulting ranked gene vector was input to a gene set enrichment analysis (GSEA) using the *fgseaMultilevel()* approach (minSize = 15, maxSize = 500). The GO, REACTOME and HALLMARK gene set collection from MsigDB were used for the GSEA (see **Tab.S7**). Enriched terms with and FDR of 0.05 were considered significant and visualized.

Single sample gene set enrichment analysis

To investigate gene set enrichment patterns per cell type and sample on pseudobulk objects, the SSGSEA approach from the GSVA package was used with default settings (except setting kcdf = “Poisson”, min.sz = 10, max.sz = 900). Mean signature scores were visualized for the healthy controls, MM responders and MM non-responders.

Pseudotime analysis

To model the NK-cell subset along the differentiation pattern from $CD56^{bright}/CD16^{dim}$ to $CD56^{dim}/CD16^{bright}$, pseudotime analysis was performed using the monocle package.[9] The NK single-cell object was converted into a monocle object and size factors and dispersion were estimated. The marker transcripts (first 200 markers per NK-cell subtype) were provided to the *setOrderingFilter()* function. After discriminative dimensionality reduction via learning a tree (DDRTree), cells were ordered along the inferred trajectory. Monocle’s intrinsic branched expression analysis modelling was used to determine genes that differ along the main pseudotime branch. Those genes that showed strong differences along the main trajectory were used for hypergeometric overrepresentation analysis via the *enricher()* function from the ClusterProfiler package utilizing the HALLMARK gene set collection.

References

1 Enßle JC, Campe J, Schwenger A, Wiercinska E, Hellstern H, Dürrwald R *et al.* Severe impairment of T-cell responses to BNT162b2 immunization in patients with multiple myeloma. *Blood* 2022; **139**: 137–142.

2 Enssle JC, Campe J, Büchel S, Moter A, See F, Grießbaum K *et al.* Enhanced but variant-dependent serological and cellular immune responses to third-dose BNT162b2 vaccination in patients with multiple myeloma. *Cancer Cell* 2022. doi:10.1016/J.CCELL.2022.05.003.

3 Amezquita RA, Lun ATL, Becht E, Carey VJ, Carpp LN, Geistlinger L *et al.* Orchestrating single-cell analysis with Bioconductor. *Nat Methods* 2020; **17**: 137–145.

4 Argelaguet R, Arnol D, Bredikhin D, Deloro Y, Velten B, Marioni JC *et al.* MOFA+: a statistical framework for comprehensive integration of multi-modal single-cell data. *Genome Biol* 2020; **21**: 111.

5 Traag VA, Waltman L, van Eck NJ. From Louvain to Leiden: guaranteeing well-connected communities. *Scientific Reports 2019 9:1* 2019; **9**: 1–12.

6 Domínguez Conde C, Xu C, Jarvis LB, Rainbow DB, Wells SB, Gomes T *et al.* Cross-tissue immune cell analysis reveals tissue-specific features in humans. *Science (1979)* 2022; **376**. doi:10.1126/SCIENCE.ABL5197/SUPPL_FILE/SCIENCE.ABL5197_MDAR_REPRODUCIBILITY_CHECKLIST.PDF.

7 Triana S, Vonficht D, Jopp-Saile L, Raffel S, Lutz R, Leonce D *et al.* Single-cell proteo-genomic reference maps of the hematopoietic system enable the purification and massive profiling of precisely defined cell states. *Nature Immunology 2021 22:12* 2021; **22**: 1577–1589.

8 Dann E, Henderson NC, Teichmann SA, Morgan MD, Marioni JC. Differential abundance testing on single-cell data using k-nearest neighbor graphs. *Nature Biotechnology 2021 40:2* 2021; **40**: 245–253.

9 Trapnell C, Cacchiarelli D, Grimsby J, Pokharel P, Li S, Morse M *et al.* The dynamics and regulators of cell fate decisions are revealed by pseudotemporal ordering of single cells. *Nature Biotechnology 2014 32:4* 2014; **32**: 381–386.

SUPPLEMENTAL FIGURES

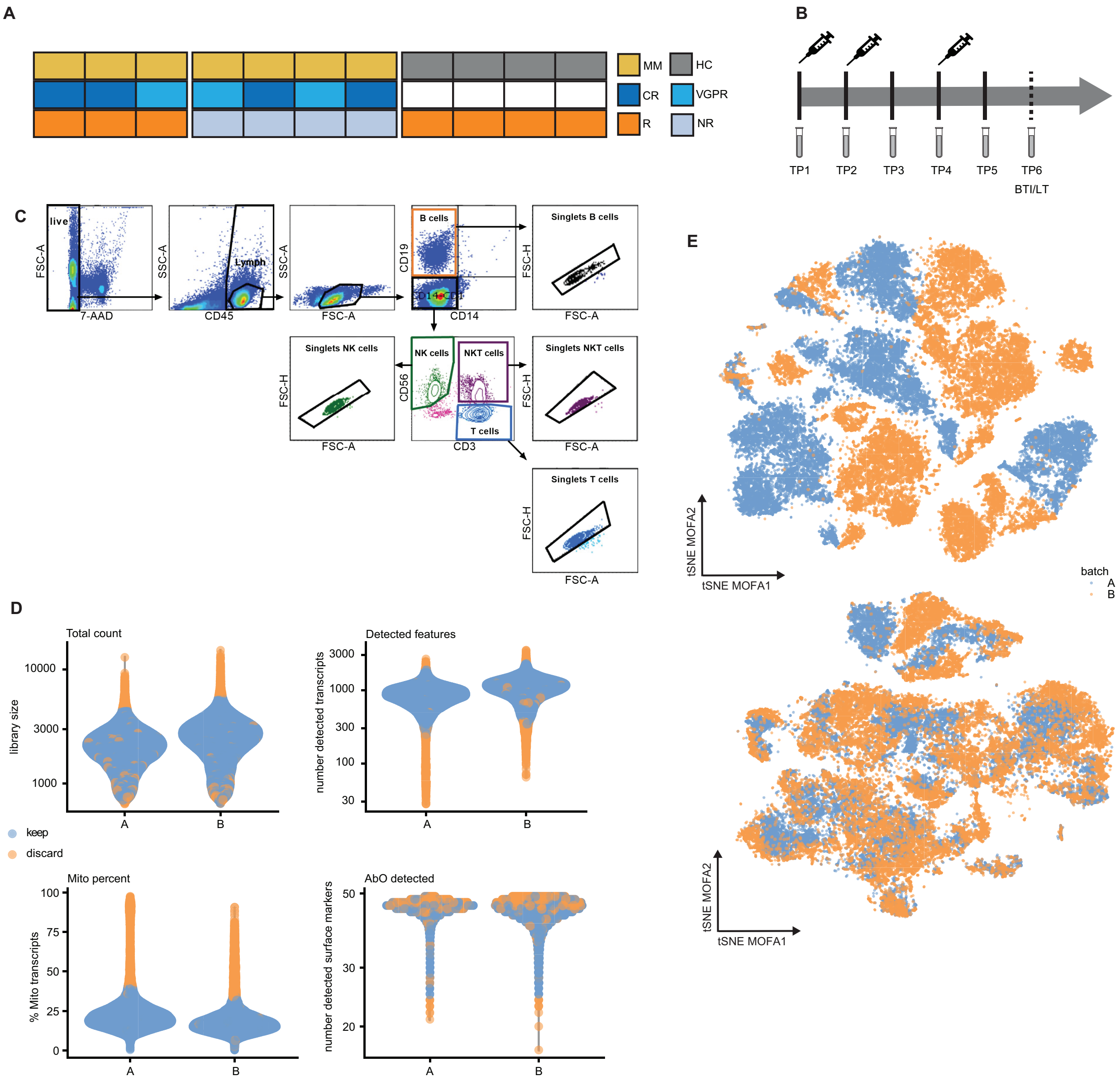

Supplementary Figure 1

Fig. S1. **Peripheral immune cell phenotypes determined by scCITEseq in patients with MM undergoing SARS-CoV-2 vaccination and breakthrough infection**

(**A**) Schematic representation of donor status regarding Multiple Myeloma (MM, yellow) vs. healthy controls (HC, grey), MM disease status (complete response, CR, dark blue; very good partial response, VGPR, light blue) and vaccination response status after 3rd vaccination (responder, R, orange; non-responder, NR, very light blue). Donors were selected if they were in the 1st line of treatment for MM, in at least very-good-partial remission (VGPR) and without corticosteroid treatment 14 days before and after vaccination. (**B**) Schematic overview about sampling timepoints. In the present study, samples obtained after 2nd vaccination (only scCITEseq), after 3rd vaccination (scCITEseq & functional readout) and at long-term (LT) or breakthrough infection (BTI) after 3rd vaccination (scCITEseq & functional readout) were analyzed. (**C**) Gating strategy for flow cytometry-based cell sorting of PBMC isolates. (**D**) Kept (blue) or removed (blue) cells after quality control for library size (upper left), number of detected features (upper right), percentage of mito transcripts (lower left) and number of detected surface markers (AbO, lower right). (**E**) T-distributed stochastic neighbour embedding (t-SNE) dimensionality reduction of MOFA-inferred latent factor before (upper) and after (lower) removal of latent-factors associating with batch vector. Abbreviations: CM, central memory; cyt, cytotoxic; EM, effector memory, M, memory; MAT, mature, N, naïve; reg, regulatory.

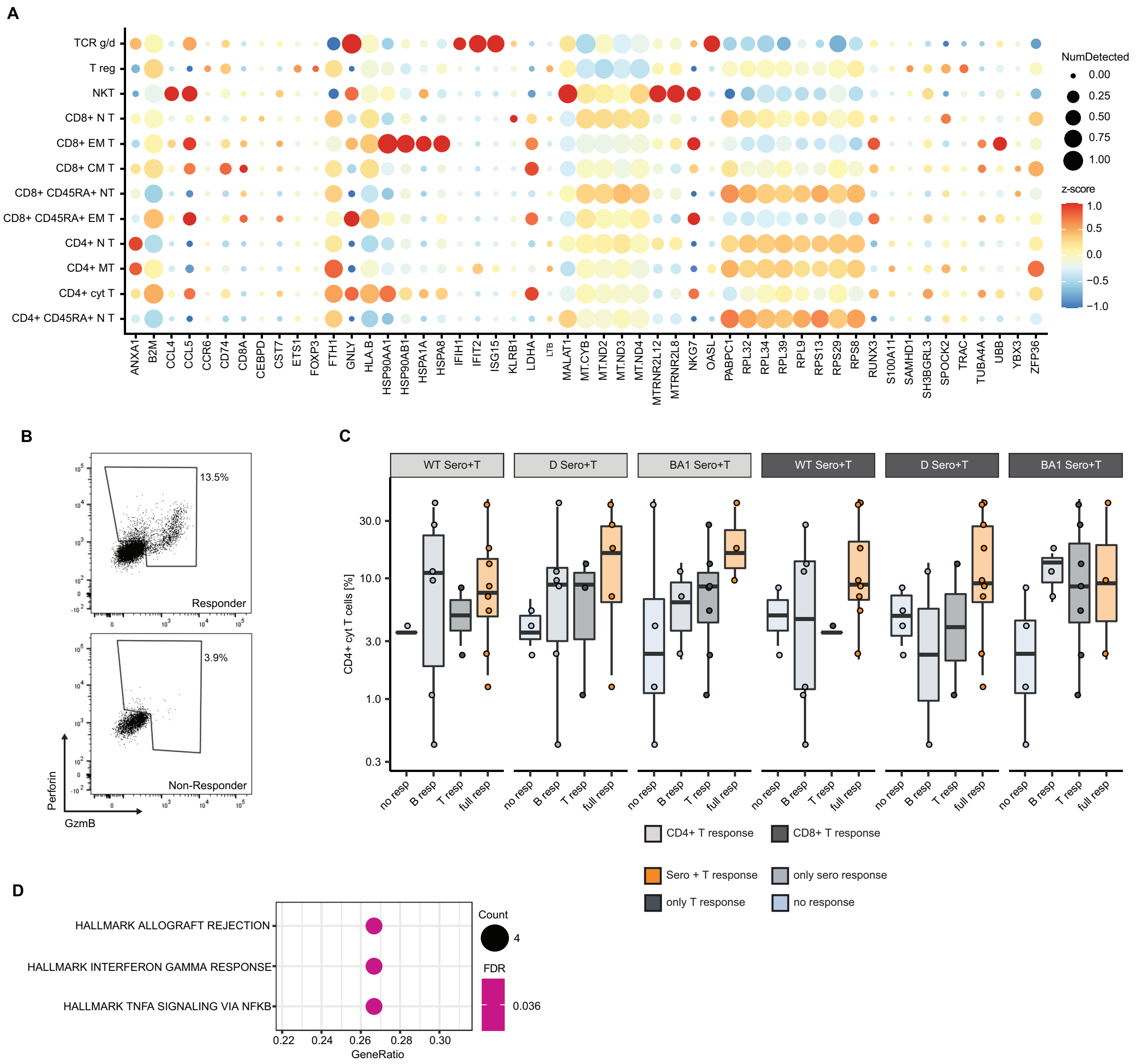

246 **Supplementary Figure 2**

Fig. S2. Cytokine-responsive T-cell populations in MM vaccination responder

(**A**) Transcriptomic markers of inferred T-cell subtypes. (**B**) Exemplary FACS Plot for $CD4^+$ cytotoxic T-cell frequencies compared between serologic responder and non-responder after the 3rd vaccination. (**C**) Frequencies of $CD4^+$ cytotoxic T cells visualized for response status (non-response, light blue; serologic response only, B resp, light grey; T-cell response only, T resp, dark grey; serologic and T-cell responder, full resp, orange) and stratified for variant (WT, wild type; D, Delta; BA1, Omicron BA.1) and $CD4^+$ (lighter grey) or $CD8^+$ (darker grey) T-cell response. $CD4^+$-WT-no resp n=1, $CD4^+$-WT-B resp n=6, $CD4^+$-WT-T resp n=2, $CD4^+$-WT-full resp n=8; $CD8^+$-WT-no resp n=2, $CD8^+$-WT-B resp n=6, $CD8^+$-WT-T resp n=1, $CD8^+$-WT-full resp n=8; $CD4^+$-D-no resp n=2, $CD4^+$-D-B resp n=6, $CD4^+$-D-T resp n=3, $CD4^+$-D-full resp n=5; $CD8^+$-D-no resp n=4, $CD8^+$-D-B resp n=2, $CD8^+$-D-T resp n=2, $CD8^+$-D-full resp n=9; $CD4^+$-BA1-no resp n=4, $CD4^+$-BA1-B resp n=3, $CD4^+$-BA1-T resp n=7, $CD4^+$-BA1-full resp n=3; $CD8^+$-BA1-no resp n=4, $CD8^+$-BA1-B resp n=3, $CD8^+$-BA1-T resp n=7, $CD8^+$-BA1-full resp n=3 (**D**) Gene set (Hallmark database) overrepresentation analysis results for inferred marker genes of T-cell neighbourhoods identified to be overrepresented in vaccination responder. Abbreviations: CM, central memory; cyt, cytotoxic; EM, effector memory, M, memory; MAT, mature, N, naïve; reg, regulatory.

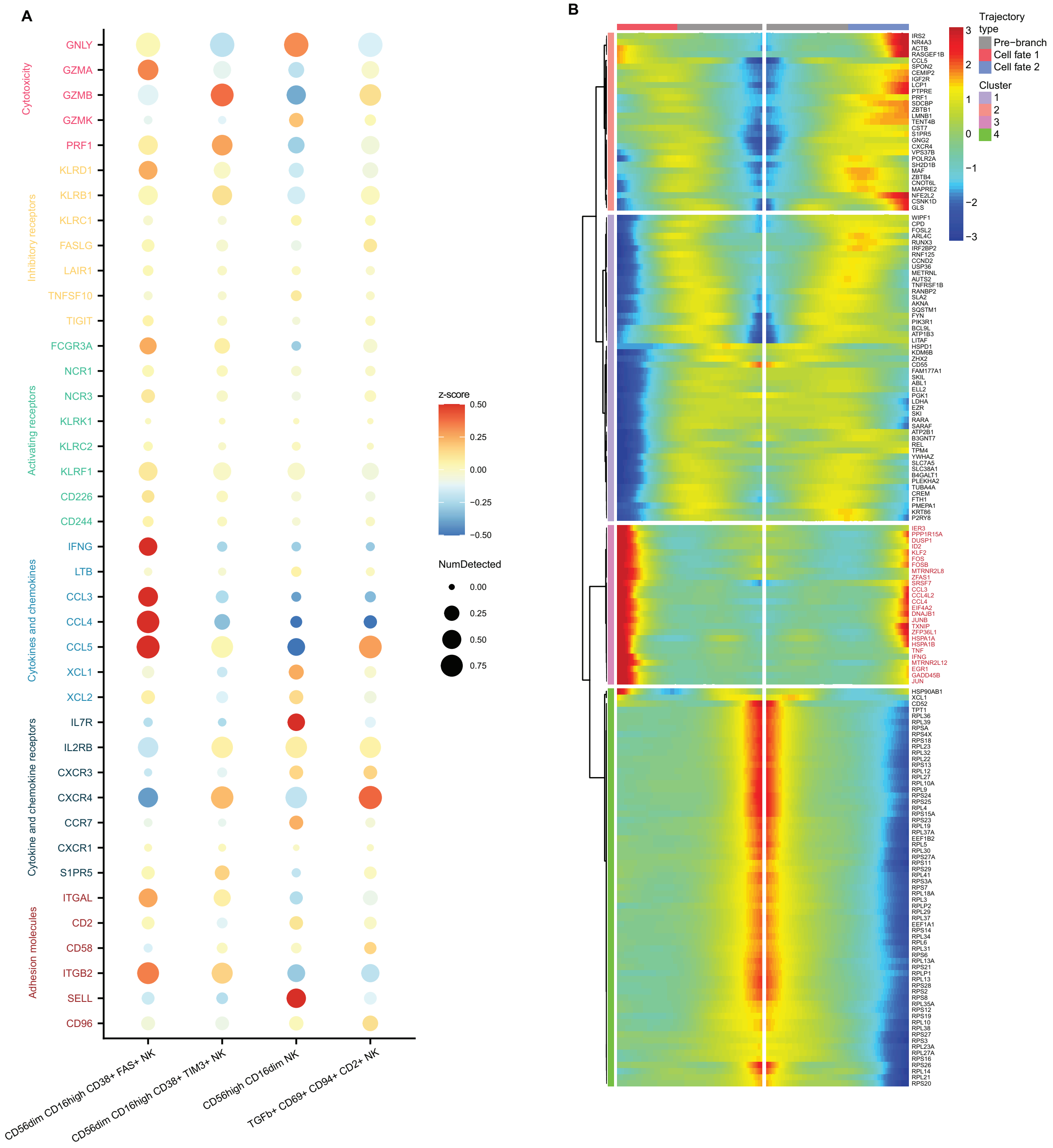

Supplementary Figure 3

Fig. S3. Functional markers of NK-cell populations and differential transcripts along NK pseudotime trajectory

(**A**) Visualization of transcript expression according to functional annotation from Smith et al, Blood Adv, 2020. (**B**) Visualization of monocle's branched expression analysis modelling results. Rows represent genes and columns points along pseudotime. The middle represents the beginning of the pseudotime and left and right border denote branches of the trajectory. Transcripts highlighted in red show increased expression in the late pseudotime branch (cell fate 1, overrepresentation of $CD56^{dim}$ $CD16^{high}$ $CD38^{+}$ FAS^{+} NK-cells).

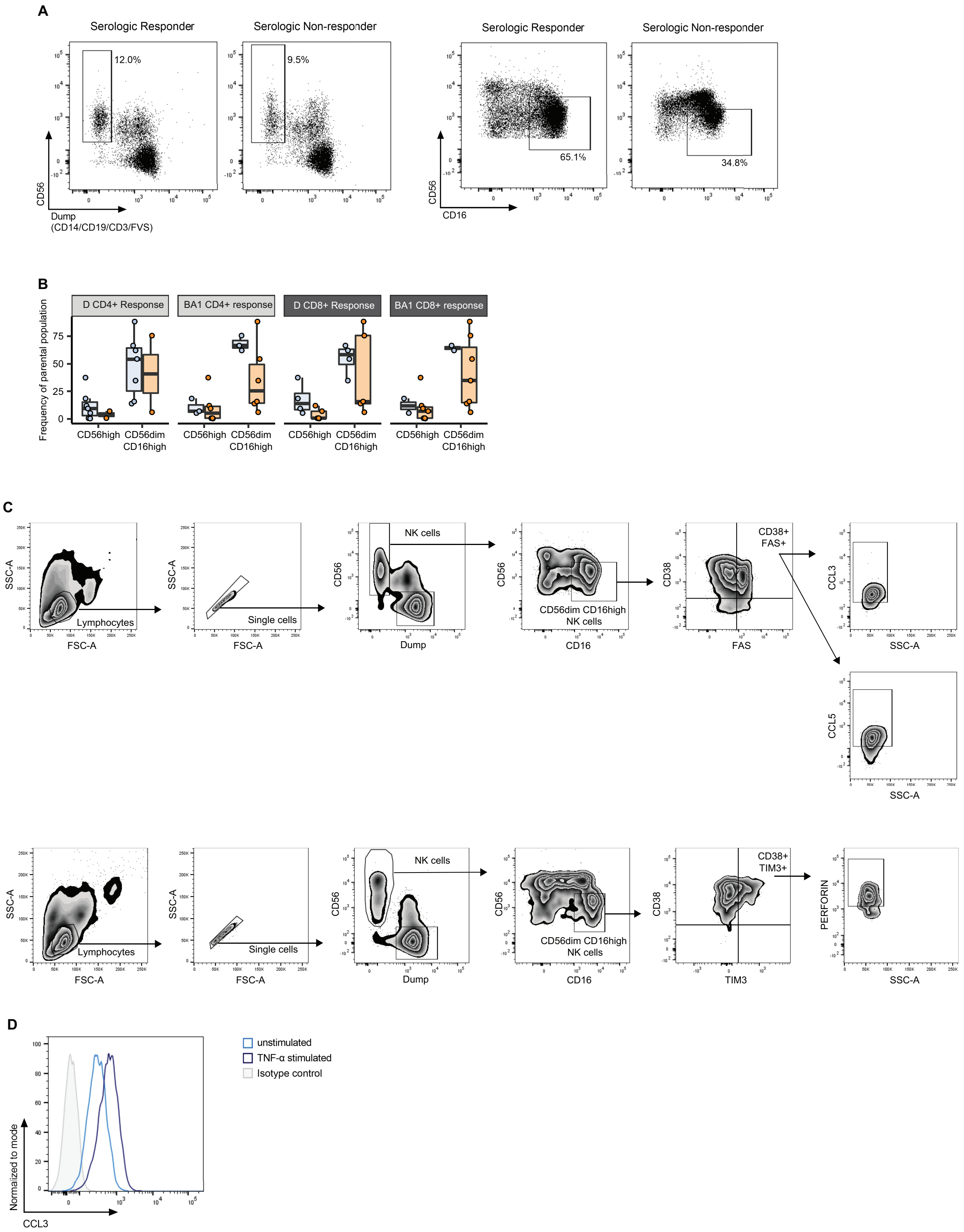

Supplementary Figure 4

Fig. S4. Cytokine-responsive mature NK-cell populations in MM vaccination responders

(**A**) Exemplary FACS plots for either CD56^{high} NK-cells (left part) or CD56^{dim} CD16^{high} NK-cells (right part) stratified for serologic responder or non-responder status. (**B**) Frequencies of highlighted NK-cell populations stratified for either T-cell non-responder (light blue) or responder (blue) for different variants (D, Delta; BA1, Omicron BA.1) and CD4^{+} (light grey) or CD8^{+} (dark grey) T-cell response. For the individual n of donors per condition, see Tab.S5 (**C**) Exemplary FACS plots for the gating strategy and recovering of CD56^{dim} CD16^{high} CD38^{+} TIM3^{+} (with additional perforin expression) or CD56^{dim} CD16^{high} CD38^{+} FAS^{+} (with additional CCL3 or CCL5 expression). (**D**) Shift in CCL3 mean fluorescence intensity without (unstimulated, blue) or after TNF-α stimulation (purple).

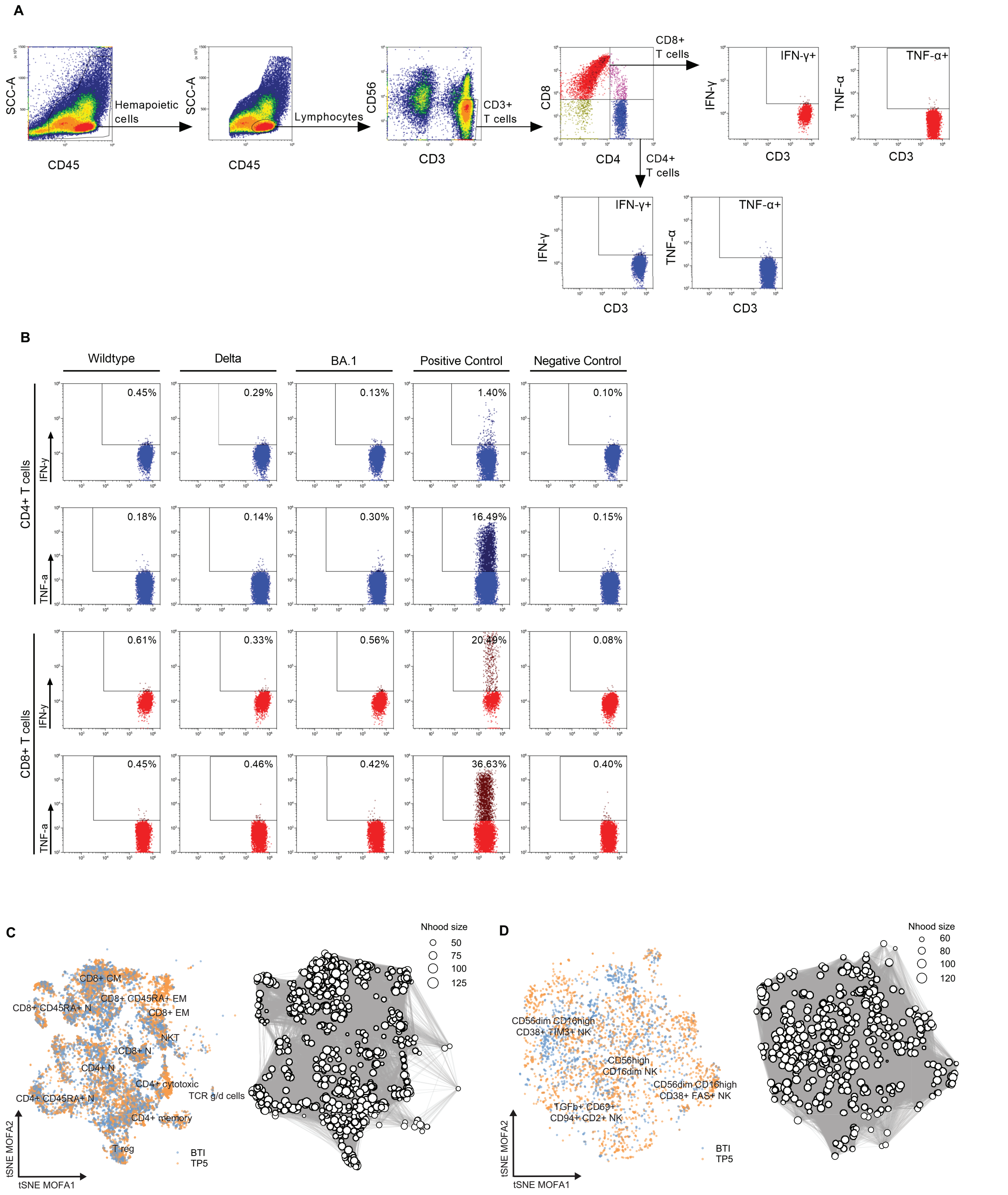

Supplementary Figure 5

Fig. S5. Cellular response after BTI in patients with MM

(**A**) Gating strategy for frequencies of SARS-CoV-2 specific und cytokine-positive T-cells. (**B**) Exemplary FACS plots for frequencies of SARS-CoV-2 specific $CD4^+$ (blue) or $CD8^+$ (red) T-cells after stimulation with the indicated peptides (WT, wild type; D, Delta; BA1, Omicron BA.1. (**C**) Differential abundance testing with miloR framework for BTI vs post 3rd timepoint (TP5) in T-cell sub-populations. (**D**) Differential abundance testing with miloR framework for BTI vs post 3rd timepoint (TP5) in NK-cell sub-populations. Abbreviations: BTI, breakthrough infection, CM, central memory; cyt, cytotoxic; EM, effector memory, M, memory; MAT, mature, N, naïve; Nhood, neighbourhood; reg, regulatory.

SUPPLEMENTAL TABLES

Table S1: Patient characteristics observation cohort

	Overall	no infection	pre 3rd	post 3rd	p-value
N patients, n (%)	105 (100.0)	53 (50.5)	15 (14.3)	37 (35.2)	
Female sex, n (%)	53 (50.5)	26 (49.1)	8 (53.3)	19 (51.4)	0.95
Age, median [IQR]	67.00 [60.00, 73.00]	68.00 [63.00, 73.00]	65.00 [55.00, 72.50]	67.00 [59.00, 75.00]	0.522
Type of MM, n (%)					0.484
IgG	50 (47.6)	24 (45.3)	9 (60.0)	17 (45.9)	
IgA	30 (28.6)	16 (30.2)	4 (26.7)	10 (27.0)	
LC	20 (19.0)	8 (15.1)	2 (13.3)	10 (27.0)	
Smouldering	4 (3.8)	4 (7.5)	0 (0.0)	0 (0.0)	
asecretoric	1 (1.0)	1 (1.9)	0 (0.0)	0 (0.0)	
ISS, n(%)					0.727
1	39 (37.1)	16 (30.2)	5 (33.3)	18 (48.6)	
2	25 (23.8)	13 (24.5)	4 (26.7)	8 (21.6)	
3	29 (27.6)	17 (32.1)	4 (26.7)	8 (21.6)	
NA	12 (11.4)	7 (13.2)	2 (13.3)	3 (8.1)	
Revised ISS, n (%)					0.114
1	28 (26.7)	14 (26.4)	3 (20.0)	11 (29.7)	
2	37 (35.2)	13 (24.5)	7 (46.7)	17 (45.9)	
3	15 (14.3)	12 (22.6)	2 (13.3)	1 (2.7)	
NA	25 (23.8)	14 (26.4)	3 (20.0)	8 (21.6)	
Genomic high risk, n (%)					0.633
no	58 (55.2)	30 (56.6)	10 (66.7)	18 (48.6)	
yes	26 (24.8)	11 (20.8)	3 (20.0)	12 (32.4)	
NA	21 (20.0)	12 (22.6)	2 (13.3)	7 (18.9)	
Current therapy line at TP5, n (%)					0.191
0	6 (5.7)	5 (9.4)	1 (6.7)	0 (0.0)	
1	61 (58.1)	23 (43.4)	11 (73.3)	27 (73.0)	
2	16 (15.2)	11 (20.8)	1 (6.7)	4 (10.8)	
3	10 (9.5)	7 (13.2)	0 (0.0)	3 (8.1)	
4	7 (6.7)	4 (7.5)	1 (6.7)	2 (5.4)	
5	2 (1.9)	2 (3.8)	0 (0.0)	0 (0.0)	
6	2 (1.9)	1 (1.9)	1 (6.7)	0 (0.0)	
9	1 (1.0)	0 (0.0)	0 (0.0)	1 (2.7)	
Remission state at TP5, n (%)					0.179
CR/VGPR	70 (66.7)	33 (62.3)	11 (73.3)	26 (70.3)	
PR	10 (9.5)	3 (5.7)	1 (6.7)	6 (16.2)	
SD/PD	25 (23.8)	17 (32.1)	3 (20.0)	5 (13.5)	
Number of HDCT, n (%)					0.582
0	33 (31.4)	19 (35.8)	4 (26.7)	10 (27.0)	

1	43 (41.0)	19 (35.8)	9 (60.0)	15 (40.5)	
2	26 (24.8)	14 (26.4)	2 (13.3)	10 (27.0)	
3	3 (2.9)	1 (1.9)	0 (0.0)	2 (5.4)	
Time since last HDCT at TP5, months [IQR]	26.00 [15.00, 52.25]	24.00 [12.25, 57.50]	26.00 [20.50, 40.50]	36.00 [16.50, 52.50]	0.714
Therapy status at TP5, n (%)					0.299
active therapy	37 (35.2)	19 (35.8)	3 (20.0)	15 (40.5)	
maintenance	33 (31.4)	13 (24.5)	7 (46.7)	13 (35.1)	
no therapy	35 (33.3)	21 (39.6)	5 (33.3)	9 (24.3)	
Current imid-based therapy at TP5, n (%)	57 (54.3)	25 (47.2)	9 (60.0)	23 (62.2)	0.332
Current PI-based therapy at TP5 n (%)	16 (15.2)	7 (13.2)	2 (13.3)	7 (18.9)	0.741
Current antiCD38-based therapy at TP5, n (%)	26 (24.8)	13 (24.5)	2 (13.3)	11 (29.7)	0.462
Basis immunization, n (%)					0.203
COMIRNATY	92 (87.6)	44 (83.0)	14 (93.3)	34 (91.9)	
VAXZEVRIA	8 (7.6)	6 (11.3)	0 (0.0)	2 (5.4)	
VAXZEVRIA+COMIRNATY	2 (1.9)	2 (3.8)	0 (0.0)	0 (0.0)	
SPIKEVAX	1 (1.0)	0 (0.0)	1 (6.7)	0 (0.0)	
COMIRNATY+SPIKEVAX	1 (1.0)	0 (0.0)	0 (0.0)	1 (2.7)	
VAXZEVRIA+SPIKEVAX	1 (1.0)	1 (1.9)	0 (0.0)	0 (0.0)	
Boost immunization, n (%)					0.412
COMIRNATY	97 (92.4)	49 (92.5)	15 (100.0)	33 (89.2)	
SPIKEVAX	8 (7.6)	4 (7.5)	0 (0.0)	4 (10.8)	

Tab. S1: Patient characteristics observation cohort

Includes all relevant disease- and patient characteristics of all patients within the observation cohort stratified for BTI infection status (no infection, pre 3rd vaccination BTI and post 3rd vaccination BTI). Abbreviations: CR, complete remission; HDCT, high-dose chemotherapy; IQR, inter-quartile range; ISS, international severity score; LC, light chain; MM, multiple myeloma; NA, not annotated; PD, progressive disease; PI, proteasome inhibitor; PR, partial remission; SD, stable disease; VGPR, very good partial remission.

Table S2: Donors per Condition SARS-CoV-2 specific T-cells (Fig. 4B)

A: CD4+ T-cells

Cytokine	Variant	Timepoint	N Donors
IFN	BA1	LT	7
IFN	BA1	post 3rd	5
IFN	BA1	pre 3rd	5
IFN	BA1	TP5	17
IFN	Delta	LT	7
IFN	Delta	post 3rd	5
IFN	Delta	pre 3rd	5
IFN	Delta	TP5	17
IFN	WT	LT	7
IFN	WT	post 3rd	5
IFN	WT	pre 3rd	5
IFN	WT	TP5	17
TNFa	BA1	LT	7
TNFa	BA1	post 3rd	5
TNFa	BA1	pre 3rd	5
TNFa	BA1	TP5	17
TNFa	Delta	LT	7
TNFa	Delta	post 3rd	5
TNFa	Delta	pre 3rd	5
TNFa	Delta	TP5	17
TNFa	WT	LT	7
TNFa	WT	post 3rd	5
TNFa	WT	pre 3rd	5
TNFa	WT	TP5	17

B: CD8+ T-cells

Cytokine	Variant	Timepoint	N Donors
IFN	BA1	LT	7
IFN	BA1	post 3rd	5
IFN	BA1	pre 3rd	5
IFN	BA1	TP5	17
IFN	Delta	LT	7
IFN	Delta	post 3rd	5
IFN	Delta	pre 3rd	5
IFN	Delta	TP5	17
IFN	WT	LT	7
IFN	WT	post 3rd	5

IFN	WT	pre 3rd	5
IFN	WT	TP5	17
TNFa	BA1	LT	7
TNFa	BA1	post 3rd	5
TNFa	BA1	pre 3rd	5
TNFa	BA1	TP5	17
TNFa	Delta	LT	7
TNFa	Delta	post 3rd	5
TNFa	Delta	pre 3rd	5
TNFa	Delta	TP5	17
TNFa	WT	LT	7
TNFa	WT	post 3rd	5
TNFa	WT	pre 3rd	5
TNFa	WT	TP5	17

Tab. S2: Donors per condition SARS-CoV-2 specific T-cells (relates to Fig. 4B)

Includes the distribution of donors per condition for the measurement of SARS-CoV-2 specific T-cells (Fig. 4B). Abbreviations: BA1, Omicron variant BA.1; IFN, Interferon; LT, long-term TNFa, Tumor-necrosis-factor α; WT, wild-type.

Table S3: Donors per T-cell response status (Fig. 4C)

Timepoint	T-cell subtype	Variant	Response status	N Donors
LT	CD4	BA1	noresp	3
LT	CD4	BA1	resp	4
LT	CD4	Delta	noresp	3
LT	CD4	Delta	resp	4
LT	CD4	WT	noresp	1
LT	CD4	WT	resp	6
LT	CD8	BA1	noresp	2
LT	CD8	BA1	resp	5
LT	CD8	Delta	noresp	2
LT	CD8	Delta	resp	5
LT	CD8	WT	noresp	1
LT	CD8	WT	resp	6
TP5	CD4	BA1	noresp	7
TP5	CD4	BA1	resp	10
TP5	CD4	Delta	noresp	9
TP5	CD4	Delta	resp	8
TP5	CD4	WT	noresp	7
TP5	CD4	WT	resp	10
TP5	CD8	BA1	noresp	7
TP5	CD8	BA1	resp	10
TP5	CD8	Delta	noresp	6
TP5	CD8	Delta	resp	11
TP5	CD8	WT	noresp	8
TP5	CD8	WT	resp	9
pre 3rd	CD4	BA1	resp	5
pre 3rd	CD4	Delta	noresp	2
pre 3rd	CD4	Delta	resp	3
pre 3rd	CD4	WT	noresp	1
pre 3rd	CD4	WT	resp	4
pre 3rd	CD8	BA1	noresp	2
pre 3rd	CD8	BA1	resp	3
pre 3rd	CD8	Delta	noresp	4
pre 3rd	CD8	Delta	resp	1
pre 3rd	CD8	WT	noresp	1
pre 3rd	CD8	WT	resp	4
post 3rd	CD4	BA1	noresp	1
post 3rd	CD4	BA1	resp	4
post 3rd	CD4	Delta	noresp	3

post 3rd	CD4	Delta	resp	2
post 3rd	CD4	WT	resp	5
post 3rd	CD8	BA1	noresp	2
post 3rd	CD8	BA1	resp	3
post 3rd	CD8	Delta	noresp	2
post 3rd	CD8	Delta	resp	3
post 3rd	CD8	WT	noresp	1
post 3rd	CD8	WT	resp	4

Tab. S3: Donors per T-cell response status (relates to Fig. 4C)

Includes the distribution of donors per T-cell status (Fig. 4C). Abbreviations: BA1, Omicron variant BA.1; LT, long-term; noresp, non-responder; resp, responder; WT, wild-type.

Table S4: Donors per response status (Fig. 4D)

Timepoint	T-cell subtype	Variant	Response status	N Donor
TP5	CD4	BA1	B resp	3
TP5	CD4	BA1	full resp	3
TP5	CD4	BA1	no resp	4
TP5	CD4	BA1	T resp	7
TP5	CD8	BA1	B resp	3
TP5	CD8	BA1	full resp	3
TP5	CD8	BA1	no resp	4
TP5	CD8	BA1	T resp	7
TP5	CD4	D	B resp	6
TP5	CD4	D	full resp	5
TP5	CD4	D	no resp	3
TP5	CD4	D	T resp	3
TP5	CD8	D	B resp	2
TP5	CD8	D	full resp	9
TP5	CD8	D	no resp	4
TP5	CD8	D	T resp	2
TP5	CD4	WT	B resp	6
TP5	CD4	WT	full resp	8
TP5	CD4	WT	no resp	1
TP5	CD4	WT	T resp	2
TP5	CD8	WT	B resp	6
TP5	CD8	WT	full resp	8
TP5	CD8	WT	no resp	2
TP5	CD8	WT	T resp	1
LT	CD4	BA1	no resp	3
LT	CD4	BA1	T resp	4
LT	CD8	BA1	no resp	2
LT	CD8	BA1	T resp	5
LT	CD4	D	B resp	1
LT	CD4	D	full resp	1
LT	CD4	D	no resp	2
LT	CD4	D	T resp	3
LT	CD8	D	B resp	1
LT	CD8	D	full resp	1
LT	CD8	D	no resp	1
LT	CD8	D	T resp	4
LT	CD4	WT	full resp	4
LT	CD4	WT	no resp	1

LT	CD4	WT	T resp	2
LT	CD8	WT	B resp	1
LT	CD8	WT	full resp	3
LT	CD8	WT	T resp	3
pre 3rd	CD4	BA1	full resp	1
pre 3rd	CD4	BA1	no resp	1
pre 3rd	CD4	BA1	T resp	3
pre 3rd	CD8	BA1	full resp	1
pre 3rd	CD8	BA1	no resp	2
pre 3rd	CD8	BA1	T resp	2
pre 3rd	CD4	D	B resp	2
pre 3rd	CD4	D	full resp	2
pre 3rd	CD4	D	no resp	1
pre 3rd	CD8	D	B resp	3
pre 3rd	CD8	D	full resp	1
pre 3rd	CD8	D	no resp	1
pre 3rd	CD4	WT	B resp	1
pre 3rd	CD4	WT	full resp	4
pre 3rd	CD8	WT	B resp	1
pre 3rd	CD8	WT	full resp	4
post 3rd	CD4	BA1	full resp	2
post 3rd	CD4	BA1	no resp	1
post 3rd	CD4	BA1	T resp	2
post 3rd	CD8	BA1	B resp	1
post 3rd	CD8	BA1	full resp	1
post 3rd	CD8	BA1	no resp	1
post 3rd	CD8	BA1	T resp	2
post 3rd	CD4	D	B resp	2
post 3rd	CD4	D	full resp	1
post 3rd	CD4	D	no resp	1
post 3rd	CD4	D	T resp	1
post 3rd	CD8	D	B resp	1
post 3rd	CD8	D	full resp	2
post 3rd	CD8	D	no resp	1
post 3rd	CD8	D	T resp	1
post 3rd	CD4	WT	full resp	3
post 3rd	CD4	WT	T resp	2
post 3rd	CD8	WT	full resp	3
post 3rd	CD8	WT	no resp	1
post 3rd	CD8	WT	T resp	1

Tab. S4: Donors per vaccination response status (relates to Fig. 4D)

Includes the distribution of donors per vaccination response status (Fig. 4D). Abbreviations: BA1, Omicron variant BA.1; B resp, serological responder; D, Delta variant; LT, long-term; resp, responder; T resp, T-cell responder; WT, wild-type.

Table S5: Donors per conditions NK cell populations (Fig. S4B)

T-cell subtype	Variant	CD	T-cell response status	N Donor
CD4	BA1	CD16pos/CD56dim	noresp	3
CD4	BA1	CD16pos/CD56dim	resp	6
CD4	BA1	CD56pos	noresp	3
CD4	BA1	CD56pos	resp	6
CD4	Delta	CD16pos/CD56dim	noresp	7
CD4	Delta	CD16pos/CD56dim	resp	2
CD4	Delta	CD56pos	noresp	7
CD4	Delta	CD56pos	resp	2
CD8	BA1	CD16pos/CD56dim	noresp	2
CD8	BA1	CD16pos/CD56dim	resp	7
CD8	BA1	CD56pos	noresp	2
CD8	BA1	CD56pos	resp	7
CD8	Delta	CD16pos/CD56dim	noresp	4
CD8	Delta	CD16pos/CD56dim	resp	5
CD8	Delta	CD56pos	noresp	4
CD8	Delta	CD56pos	resp	5

Tab. S5: Donors per condition NK-cell populations (relates to Fig. S4B)

Includes the distribution of donors per condition of NK-cell populations (Fig. S4B). Abbreviations: BA1, Omicron variant BA.1; D, Delta variant; LT, long-term; noresp, non-responder; resp, responder; WT, wild-type.

Table S6: Sorted cells per Donor and condition for scCITEseq

Donor	ID	Timepoint	Group	Status	Subtype	Number of cells sorted (x10E3)	Batch
Donor1	Donor1_TP3_MM_MM3	TP3 (post 2nd)	Ser- T cell-	MM	T cells	30.000	MM3
Donor1	Donor1_TP3_MM_MM3	TP3 (post 2nd)	Ser- T cell-	MM	NK cells	6.293	MM3
Donor1	Donor1_TP3_MM_MM3	TP3 (post 2nd)	Ser- T cell-	MM	NKT cells	5.000	MM3
Donor1	Donor1_TP3_MM_MM3	TP3 (post 2nd)	Ser- T cell-	MM	B cells	10.000	MM3
Donor1	Donor1_TP5_MM_MM3	TP5 (post 3rd)	Ser- T cell-	MM	T cells	30.000	MM3
Donor1	Donor1_TP5_MM_MM3	TP5 (post 3rd)	Ser- T cell-	MM	NK cells	3.111	MM3
Donor1	Donor1_TP5_MM_MM3	TP5 (post 3rd)	Ser- T cell-	MM	NKT cells	5.000	MM3
Donor1	Donor1_TP5_MM_MM3	TP5 (post 3rd)	Ser- T cell-	MM	B cells	2.806	MM3
Donor2	Donor2_TP3_MM_MM3	TP3 (post 2nd)	Ser- T cell-	MM	T cells	30.000	MM3
Donor2	Donor2_TP3_MM_MM3	TP3 (post 2nd)	Ser- T cell-	MM	NK cells	7.478	MM3
Donor2	Donor2_TP3_MM_MM3	TP3 (post 2nd)	Ser- T cell-	MM	NKT cells	5.000	MM3
Donor2	Donor2_TP3_MM_MM3	TP3 (post 2nd)	Ser- T cell-	MM	B cells	6.273	MM3
Donor2	Donor2_TP5_MM_MM3	TP5 (post 3rd)	Ser- T cell-	MM	T cells	30.000	MM3
Donor2	Donor2_TP5_MM_MM3	TP5 (post 3rd)	Ser- T cell-	MM	NK cells	10.000	MM3
Donor2	Donor2_TP5_MM_MM3	TP5 (post 3rd)	Ser- T cell-	MM	NKT cells	2.738	MM3
Donor2	Donor2_TP5_MM_MM3	TP5 (post 3rd)	Ser- T cell-	MM	B cells	10.000	MM3
Donor3	Donor3_TP3_MM_MM3	TP3 (post 2nd)	Ser+ T cell+	MM	T cells	30.000	MM3
Donor3	Donor3_TP3_MM_MM3	TP3 (post 2nd)	Ser+ T cell+	MM	NK cells	10.000	MM3
Donor3	Donor3_TP3_MM_MM3	TP3 (post 2nd)	Ser+ T cell+	MM	NKT cells	5.000	MM3
Donor3	Donor3_TP3_MM_MM3	TP3 (post 2nd)	Ser+ T cell+	MM	B cells	10.000	MM3
Donor3	Donor3_TP5_MM_MM3	TP5 (post 3rd)	Ser+ T cell+	MM	T cells	30.000	MM3
Donor3	Donor3_TP5_MM_MM3	TP5 (post 3rd)	Ser+ T cell+	MM	NK cells	10.000	MM3
Donor3	Donor3_TP5_MM_MM3	TP5 (post 3rd)	Ser+ T cell+	MM	NKT cells	5.000	MM3
Donor3	Donor3_TP5_MM_MM3	TP5 (post 3rd)	Ser+ T cell+	MM	B cells	10.000	MM3
Donor4	Donor4_TP3_MM_MM3	TP3 (post 2nd)	Ser+ T cell+	MM	T cells	30.000	MM3
Donor4	Donor4_TP3_MM_MM3	TP3 (post 2nd)	Ser+ T cell+	MM	NK cells	10.000	MM3
Donor4	Donor4_TP3_MM_MM3	TP3 (post 2nd)	Ser+ T cell+	MM	NKT cells	5.000	MM3
Donor4	Donor4_TP3_MM_MM3	TP3 (post 2nd)	Ser+ T cell+	MM	B cells	10.000	MM3
Donor4	Donor4_TP5_MM_MM3	TP5 (post 3rd)	Ser+ T cell+	MM	T cells	30.000	MM3
Donor4	Donor4_TP5_MM_MM3	TP5 (post 3rd)	Ser+ T cell+	MM	NK cells	10.000	MM3
Donor4	Donor4_TP5_MM_MM3	TP5 (post 3rd)	Ser+ T cell+	MM	NKT cells	5.000	MM3
Donor4	Donor4_TP5_MM_MM3	TP5 (post 3rd)	Ser+ T cell+	MM	B cells	10.000	MM3
Donor5	Donor5_TP3_Ctrl_MM3	TP3 (post 2nd)	Control	HC	T cells	30.000	MM3
Donor5	Donor5_TP3_Ctrl_MM3	TP3 (post 2nd)	Control	HC	NK cells	10.000	MM3
Donor5	Donor5_TP3_Ctrl_MM3	TP3 (post 2nd)	Control	HC	NKT cells	3.065	MM3
Donor5	Donor5_TP3_Ctrl_MM3	TP3 (post 2nd)	Control	HC	B cells	10.000	MM3
Donor5	Donor5_TP5_Ctrl_MM3	TP5 (post 3rd)	Control	HC	T cells	30.000	MM3
Donor5	Donor5_TP5_Ctrl_MM3	TP5 (post 3rd)	Control	HC	NK cells	10.000	MM3

Donor5	Donor5_TP5_Ctrl_MM3	TP5 (post 3rd)	Control	HC	NKT cells	5.000	MM3
Donor5	Donor5_TP5_Ctrl_MM3	TP5 (post 3rd)	Control	HC	B cells	10.000	MM3
Donor6	Donor6_TP3_Ctrl_MM3	TP3 (post 2nd)	Control	HC	T cells	30.000	MM3
Donor6	Donor6_TP3_Ctrl_MM3	TP3 (post 2nd)	Control	HC	NK cells	10.000	MM3
Donor6	Donor6_TP3_Ctrl_MM3	TP3 (post 2nd)	Control	HC	NKT cells	5.000	MM3
Donor6	Donor6_TP3_Ctrl_MM3	TP3 (post 2nd)	Control	HC	B cells	10.000	MM3
Donor6	Donor6_TP5_Ctrl_MM3	TP5 (post 3rd)	Control	HC	T cells	30.000	MM3
Donor6	Donor6_TP5_Ctrl_MM3	TP5 (post 3rd)	Control	HC	NK cells	10.000	MM3
Donor6	Donor6_TP5_Ctrl_MM3	TP5 (post 3rd)	Control	HC	NKT cells	5.000	MM3
Donor6	Donor6_TP5_Ctrl_MM3	TP5 (post 3rd)	Control	HC	B cells	10.000	MM3
Donor7	Donor7_TP5_MM_MM4	TP5 (post 3rd)	Ser- T cell-	MM	T cells	30.000	MM4
Donor7	Donor7_TP5_MM_MM4	TP5 (post 3rd)	Ser- T cell-	MM	NK cells	10.000	MM4
Donor7	Donor7_TP5_MM_MM4	TP5 (post 3rd)	Ser- T cell-	MM	NKT cells	5.000	MM4
Donor7	Donor7_TP5_MM_MM4	TP5 (post 3rd)	Ser- T cell-	MM	B cells	10.000	MM4
Donor7	Donor7_Breakthrough_MM_MM4	BTI post 3rd	Ser- T cell-	MM	T cells	30.000	MM4
Donor7	Donor7_Breakthrough_MM_MM4	BTI post 3rd	Ser- T cell-	MM	NK cells	4.266	MM4
Donor7	Donor7_Breakthrough_MM_MM4	BTI post 3rd	Ser- T cell-	MM	NKT cells	5.000	MM4
Donor7	Donor7_Breakthrough_MM_MM4	BTI post 3rd	Ser- T cell-	MM	B cells	10.000	MM4
Donor8	Donor8_TP5_MM_MM4	TP5 (post 3rd)	Ser- T cell-	MM	T cells	30.000	MM4
Donor8	Donor8_TP5_MM_MM4	TP5 (post 3rd)	Ser- T cell-	MM	NK cells	10.000	MM4
Donor8	Donor8_TP5_MM_MM4	TP5 (post 3rd)	Ser- T cell-	MM	NKT cells	5.000	MM4
Donor8	Donor8_TP5_MM_MM4	TP5 (post 3rd)	Ser- T cell-	MM	B cells	10.000	MM4
Donor8	Donor8_Breakthrough_MM_MM4	BTI post 3rd	Ser- T cell-	MM	T cells	30.000	MM4
Donor8	Donor8_Breakthrough_MM_MM4	BTI post 3rd	Ser- T cell-	MM	NK cells	10.000	MM4
Donor8	Donor8_Breakthrough_MM_MM4	BTI post 3rd	Ser- T cell-	MM	NKT cells	5.000	MM4
Donor8	Donor8_Breakthrough_MM_MM4	BTI post 3rd	Ser- T cell-	MM	B cells	10.000	MM4
Donor4	Donor4_TP5_MM_MM4	TP5 (post 3rd)	Ser+ T cell+	MM	T cells	30.000	MM4
Donor4	Donor4_TP5_MM_MM4	TP5 (post 3rd)	Ser+ T cell+	MM	NK cells	10.000	MM4
Donor4	Donor4_TP5_MM_MM4	TP5 (post 3rd)	Ser+ T cell+	MM	NKT cells	5.000	MM4
Donor4	Donor4_TP5_MM_MM4	TP5 (post 3rd)	Ser+ T cell+	MM	B cells	10.000	MM4
Donor4	Donor4_Breakthrough_MM_MM4	BTI post 3rd	Ser+ T cell+	MM	T cells	30.000	MM4
Donor4	Donor4_Breakthrough_MM_MM4	BTI post 3rd	Ser+ T cell+	MM	NK cells	10.000	MM4
Donor4	Donor4_Breakthrough_MM_MM4	BTI post 3rd	Ser+ T cell+	MM	NKT cells	5.000	MM4
Donor4	Donor4_Breakthrough_MM_MM4	BTI post 3rd	Ser+ T cell+	MM	B cells	10.000	MM4
Donor9	Donor9_TP5_MM_MM4	TP5 (post 3rd)	Ser+ T cell+	MM	T cells	30.000	MM4
Donor9	Donor9_TP5_MM_MM4	TP5 (post 3rd)	Ser+ T cell+	MM	NK cells	10.000	MM4
Donor9	Donor9_TP5_MM_MM4	TP5 (post 3rd)	Ser+ T cell+	MM	NKT cells	5.000	MM4
Donor9	Donor9_TP5_MM_MM4	TP5 (post 3rd)	Ser+ T cell+	MM	B cells	3.699	MM4
Donor9	Donor9_Breakthrough_MM_MM4	BTI post 3rd	Ser+ T cell+	MM	T cells	30.000	MM4
Donor9	Donor9_Breakthrough_MM_MM4	BTI post 3rd	Ser+ T cell+	MM	NK cells	10.000	MM4

Donor9	Donor9_Breakthrough_MM_MM4	BTI post 3rd	Ser+ T cell+	MM	NKT cells	3.797	MM4
Donor9	Donor9_Breakthrough_MM_MM4	BTI post 3rd	Ser+ T cell+	MM	B cells	8.571	MM4
Donor10	Donor10_TP5_Ctrl_MM4	TP5 (post 3rd)	Control	HC	T cells	30.000	MM4
Donor10	Donor10_TP5_Ctrl_MM4	TP5 (post 3rd)	Control	HC	NK cells	8.013	MM4
Donor10	Donor10_TP5_Ctrl_MM4	TP5 (post 3rd)	Control	HC	NKT cells	2.793	MM4
Donor10	Donor10_TP5_Ctrl_MM4	TP5 (post 3rd)	Control	HC	B cells	10.000	MM4
Donor10	Donor10_Breakthrough_Ctrl_MM4	BTI post 3rd	Control	HC	T cells	30.000	MM4
Donor10	Donor10_Breakthrough_Ctrl_MM4	BTI post 3rd	Control	HC	NK cells	10.000	MM4
Donor10	Donor10_Breakthrough_Ctrl_MM4	BTI post 3rd	Control	HC	NKT cells	5.000	MM4
Donor10	Donor10_Breakthrough_Ctrl_MM4	BTI post 3rd	Control	HC	B cells	10.000	MM4
Donor11	Donor11_TP5_Ctrl_MM4	TP5 (post 3rd)	Control	HC	T cells	30.000	MM4
Donor11	Donor11_TP5_Ctrl_MM4	TP5 (post 3rd)	Control	HC	NK cells	10.000	MM4
Donor11	Donor11_TP5_Ctrl_MM4	TP5 (post 3rd)	Control	HC	NKT cells	5.000	MM4
Donor11	Donor11_TP5_Ctrl_MM4	TP5 (post 3rd)	Control	HC	B cells	10.000	MM4
Donor11	Donor11_Breakthrough_Ctrl_MM4	BTI post 3rd	Control	HC	T cells	30.000	MM4
Donor11	Donor11_Breakthrough_Ctrl_MM4	BTI post 3rd	Control	HC	NK cells	10.000	MM4
Donor11	Donor11_Breakthrough_Ctrl_MM4	BTI post 3rd	Control	HC	NKT cells	5.000	MM4
Donor11	Donor11_Breakthrough_Ctrl_MM4	BTI post 3rd	Control	HC	B cells	10.000	MM4

Tab. S6: Sorted cells for donor and condition for scCITEseq

Includes the number of sorted cells (T, B, NK, NKT) per donor and condition. "." Indicates decimal separator. Abbreviations: HC, healthy control; MM, multiple myeloma, Ser +, serological responder; Ser -, serological non-responder. T cell +, T-cell responder; T cell -, T-cell non-responder.

Table S7: Materials and resources

Reagent/ Resource	Source	Identifier
Antibodies		
7AAD	BD Biosciences	Cat#559925
BB515-CD19 (clone HIBI19)	BD Biosciences	Cat#564456
BV711-CD14 (M5E2)	Biolegend	Cat#301838
BV510-CD45 (clone HI30)	BD Biosciences	Cat#563204
BV421-CD56 (clone NCAM 16.2)	BD Biosciences	Cat#562751
BUV395-CD3 (clone SK7)	BD Biosciences	Cat#564001
PerCP-CD3 (clone UCHT-1)	Biolegend	Cat#300428
BV786-CD4 (clone SK3)	BD Biosciences	Cat#563877
BUV737-CD8 (clone SK1)	BD Biosciences	Cat#612754
BV510-CD56 (NCAM16.2)	BD Biosciences	Cat#563041
BV510-CD19 (SJ25C1)	BD Biosciences	Cat#562947
BV510-CD14 (clone MφP9)	BD Biosciences	Cat#563079
BV421-Perforin (clone δG9)	Biolegend	Cat#563393
BV421-Mouse IgG2b,κ (clone 27-35)	BD Biosciences	Cat#562748
FITC-Granzyme B (clone REA226)	Miltenyi Biotec	Cat#130-118-341
BV786-CD56 (clone MY31)	BD Biosciences	Cat#742662
PE-CF594-CD16 (clone 3G8)	BD Biosciences	Cat#562293
BV510-CD3 (clone UCHT-1)	BD Biosciences	Cat#563109
BUV395-CD38 (clone HB7)	BD Biosciences	Cat#563811
BUV737-CD18 (clone mAb24)	BD Biosciences	Cat#752927
BV421-CD95 (Fas, clone DX2)	BD Biosciences	Cat#562648
BV605-CD366 (Tim-3, clone 344823)	BD Biosciences	Cat#747961
BB515-CCL5 (clone 2D5)	BD Biosciences	Cat#564752

BB515-Mouse IgG1,κ (clone X40)	BD Biosciences	Cat#564416
PE-CCL3 (clone D21-1351)	BD Biosciences	Cat#561120
PE-Mouse IgG2a,κ (clone G155-178)	BD Biosciences	Cat#554648
APC-Vio770-CD4 (clone REA623)	Miltenyi Biotec	Cat#130-113-223
PE-IFN-y (clone REA600)	Miltenyi Biotec	Cat#130-113-498
PE-Vio 770-TNF-a (clone REA656)	Miltenyi Biotec	Cat#130-127-531
BV786-CD56 (clone NCAM16.2)	BD Biosciences	Cat#564058
KrO-CD45 (clone J33)	Beckman Coulter	Cat#B36294
APC-CD3 (clone UCHT1)	Beckman Coulter	Cat#IM2467
Alexa Fluor 700-CD8 (clone B9.11)	Beckman Coulter	Cat#B76279
human IgG	Kiovig or Intratect	N/A
Fixable viability stain 510	BD Biosciences	Cat#564406
BD AbSeq Oligo Mouse Anti-Human CD3, Clone: SK7	BD Biosciences	Cat#940000
BD AbSeq Oligo Mouse Anti-Human CD4, Clone: SK3	BD Biosciences	Cat#940001
BD AbSeq Oligo Mouse Anti-Human CD19, Clone: SJ25C1	BD Biosciences	Cat#940004
BD AbSeq Oligo Mouse Anti-Human CD14, Clone: MφP9	BD Biosciences	Cat#940005
BD AbSeq Oligo Mouse Anti-Human CD56, Clone: NCAM16.2	BD Biosciences	Cat#940007

BD AbSeq Oligo Mouse Anti-Human CD25 (IL-2 Receptor α), Clone: 2A3	BD Biosciences	Cat#940009
BD AbSeq Oligo Mouse Anti-Human CD45RA, Clone: HI100	BD Biosciences	Cat#940011
BD AbSeq Oligo Mouse Anti-Human CD127, Clone: HIL-7R-M21	BD Biosciences	Cat#940012
BD AbSeq Oligo Mouse Anti-Human CD38, Clone: HIT2	BD Biosciences	Cat#940013
BD AbSeq Oligo Mouse Anti-Human CD279 (PD-1), Clone: EH12.1	BD Biosciences	Cat#940015
BD AbSeq Oligo Mouse Anti-Human CD28, Clone: CD28.2	BD Biosciences	Cat#940017
BD AbSeq Oligo Mouse Anti-Human CD69, Clone: FN50	BD Biosciences	Cat#940019
BD AbSeq Oligo Mouse Anti-Human CD34, Clone: 581	BD Biosciences	Cat#940021
BD AbSeq Oligo Mouse Anti-Human CD45RO, Clone: UCHL1	BD Biosciences	Cat#940022
BD AbSeq Oligo Mouse Anti-Human IgD, Clone: IA6-2	BD Biosciences	Cat#940026
BD AbSeq Oligo Mouse Anti-Human IgG, Clone: G18-145	BD Biosciences	Cat#940027

BD AbSeq Oligo Mouse Anti-Human CD7, Clone: M-T701	BD Biosciences	Cat#940029
BD AbSeq Oligo Mouse Anti-Human CD33, Clone: WM53	BD Biosciences	Cat#940031
BD AbSeq Oligo Mouse Anti-Human CD152, Clone: BNI3	BD Biosciences	Cat#940034
BD AbSeq Oligo Mouse Anti-Human CD95, Clone: DX2	BD Biosciences	Cat#940037
BD AbSeq Oligo Mouse Anti-Human CD5, Clone: UCHT2	BD Biosciences	Cat#940038
BD AbSeq Oligo Mouse Anti-Human CD2, Clone: RPA-2.10	BD Biosciences	Cat#940046
BD AbSeq Oligo Mouse Anti-Human CD21, Clone: B-ly4	BD Biosciences	Cat#940048
BD AbSeq Oligo Mouse Anti-Human CD40, Clone: 5c3	BD Biosciences	Cat#940049
BD AbSeq Oligo Mouse Anti-Human CD81 (TAPA-1), Clone: JS-81	BD Biosciences	Cat#940052
BD AbSeq Oligo Mouse Anti-Human CD314 (NKG2D), Clone: 1D11	BD Biosciences	Cat#940061
BD AbSeq Oligo Mouse Anti-Human CD335 (NKp46),	BD Biosciences	Cat#940064

Clone: 9E2/NKp46		
BD AbSeq Oligo Mouse Anti-Human TIM-3 (CD366), Clone: D3	BD Biosciences	Cat#940066
BD AbSeq Oligo Mouse Anti-Human CD103, Clone: Ber-ACT8	BD Biosciences	Cat#940067
BD AbSeq Oligo Mouse Anti-Human CD161, Clone: DX12	BD Biosciences	Cat#940070
BD AbSeq Oligo Mouse Anti-Human CD39, Clone: TU66	BD Biosciences	Cat#940073
BD AbSeq Oligo Mouse Anti-Human TCRαβ, Clone: IP26	BD Biosciences	Cat#940074
BD AbSeq Oligo Mouse Anti-Human CD226, Clone: DX11	BD Biosciences	Cat#940075
BD AbSeq Oligo Mouse Anti-Human CD94, Clone: HP-3D9	BD Biosciences	Cat#940081
BD AbSeq Oligo Mouse Anti-Human CD18, Clone: 45113	BD Biosciences	Cat#940086
BD AbSeq Oligo Mouse Anti-Human CD26, Clone: M-A261	BD Biosciences	Cat#940101
BD AbSeq Oligo Rat Anti-Human CX3CR1, Clone: 2A9-1	BD Biosciences	Cat#940216
BD AbSeq Oligo Mouse Anti-Human	BD Biosciences	Cat#940232

CD122, Clone: Mik-β3/IL2RB		
BD AbSeq Oligo Mouse Anti-Human CD102, Clone: CBR-IC2/2	BD Biosciences	Cat#940241
BD AbSeq Oligo Mouse Anti-Human CD11b, Clone: ICRF44	BD Biosciences	Cat#940266
BD AbSeq Oligo Mouse Anti-Human CD22, Clone: HIB22	BD Biosciences	Cat#940273
BD AbSeq Oligo Mouse Anti-Human IgM, Clone: G20-127	BD Biosciences	Cat#940276
BD AbSeq Oligo Mouse Anti-Human CD215 (IL-15Rα), Clone: JM7A4	BD Biosciences	Cat#940290
BD AbSeq Oligo Mouse Anti-Human CD1d, Clone: CD1d42	BD Biosciences	Cat#940296
BD AbSeq Oligo Mouse Anti-Human CD8, Clone: SK1	BD Biosciences	Cat#940305
BD AbSeq Oligo Mouse Anti-Human CD16, Clone: B73.1	BD Biosciences	Cat#940314
BD AbSeq Oligo Mouse Anti-Human CD27, Clone: L128	BD Biosciences	Cat#940319
BD AbSeq Oligo Mouse Anti-Human γδ TCR, Clone: 11F2	BD Biosciences	Cat#940365
BD AbSeq Oligo Mouse Anti-Human	BD Biosciences	Cat#940394

CCR7 (CD197), Clone: 2-L1-A		
Bacterial and virus strains		
SARS-CoV-2 FFM1 isolate [wild type, WT/parental]	Toptan T et al., Int J Mol Sci, 2020	GenBank ID: MT358638
SARS-CoV-2 isolates B.1.617.2 FFM-IND8424/2021 [Delta]	Wilhelm A et al., eBioMedicine, 2022	GenBank ID: MZ315141
B.1.1.529 (BA.1) FFM-SIM0550/2021 [Omicron BA.1]	Wilhelm A et al., eBioMedicine, 2022	GenBank ID: OL800702
Biological samples		
Lithium heparin whole blood from patients with MM and healthy controls	Department of Medicine II, Hematology/Oncology, University Hospital Frankfurt, Frankfurt, Germany	N/A
CACO-2 colon adenocarcinoma cell line	American Type Culture Collection, Manassas, Virginia, USA Deutsche Sammlung von Mikroorganismen und Zellkulturen	ACC-169
Chemicals, peptides, and recombinant proteins		
Dulbecco's Phosphate Buffered Saline (DPBS 1X)	Gibco	Cat#14190-094
Ampuwa, sterile water for injections	Fresenius Kabi AG	Cat#B230673
Trypan blue stain (0.4%)	Thermo Fisher Scientific	Cat#1525-061
Histopaque-1077	Sigma Aldrich	Cat#10771
RPMI1640 medium	Gibco	Cat#11530586
Fetal bovine serum	Thermo Fisher Scientific	Cat#A5256701
RPMI1640 medium, GlutaMAX Supplement	Gibco	Cat#61870036
Penicillin/Streptomycin	Gibco	Cat#15070063
Brilliant stain buffer	BD Biosciences	Cat#563794
Formaldehyde-Solution 37%	AppliChem GmbH	Cat#141328
Saponin	Sigma-Aldrich	Cat#S4521-10G

Bovine serum albumin	Sigma Life Science	Cat#A2153-100G
recombinant human TNF-a	Peprotech	Cat#300-01A
BD GolgiStop	BD Biosciences	Cat#554724
Brefeldin A Solution	Biolegend	Cat#420601
Dimethyl Sulfoxide (DMSO)	Sigma Aldrich	Cat#D2650-100ml
Cytostim	Miltenyi Biotec	Cat#130-092-173
Inside Stain Kit	Miltenyi Biotec	Cat#130-090-477
PepTivator SARS-CoV-2 Prot_S	Miltenyi Biotec	Cat#130-126-700
PepTivator SARS-CoV-2 Prot_S B.1.617.2	Miltenyi Biotec	Cat#130-128-763
PepTivator SARS-CoV-2 Prot_S B11529	Miltenyi Biotec	Cat#130-129-927
Human Interleukin 12 (IL-12)	PeproTech	Cat#200-12
Human Interleukin 15 (IL-15)	Miltenyi Biotec	Cat#130-095-765
Recombinant Human Interleukin 18 (IL-18)	MBL	Cat#B001-5
BD Stain Buffer (BSA)	BD Biosciences	Cat#554657
BD Pharmingen Calcein AM	BD Biosciences	Cat#564061
BD Pharmingen DRAQ7	BD Biosciences	Cat#564904
TapeStation High Sensitivity D1000 Reagents	Agilent	Cat#5067-5585 & #5067-5584
Critical commercial assays		
BD Rhapsody Cartridge Reagent Kit	BD Biosciences	Cat#633731
BD Rhapsody Cartridge Kit	BD Biosciences	Cat#633733
BD Rhapsody cDNA Kit	BD Biosciences	Cat#633773
BD Rhapsody Whole Transcriptome Analysis (WTA)	BD Biosciences	Cat#633801

Amplification Kit		
BD Rhapsody Single-Cell Multiplexing Kit	BD Biosciences	Cat#633781
Qubit dsDNA HS Kit	Thermo Fisher Scientific	Cat#Q32851
SARS-CoV-2-IgG, chemiluminescent microparticle immunoassay (CMIA)	Abbott	Cat#06R90
SARS-CoV-2 IgG II Quant CMIA	Abbott	Cat#06S60
Deposited data		
CITE sequencing data (whole transcriptome and Abseq data)	This publication	GSE229187
C5 gene set collection: gene ontology gene sets	Carbon et al., 2021	https://www.gsea-msigdb.org/gsea/msigdb/human/collections.jsp#C5
H gene set collection: hallmark gene sets	Liberzon et al., 2015	https://www.gsea-msigdb.org/gsea/msigdb/human/collections.jsp#H
C2 curated gene set collection : Reactome subset	Fabregat et al., 2018	https://www.gsea-msigdb.org/gsea/msigdb/human/collections.jsp#C2
Cell sort and CITEseq donor meta data	This publication	N/A
Software and algorithms		
FlowJo Software (v10.3)	BD Biosciences	N/A
Kaluza Analysis	Beckman Coulter	N/A
SevenBridges Platform	BD Biosciences	https://www.sevenbridges.com
BaseSpace Sequence Hub	Illumina	https://basespace.illumina.com
R	R Development Core Team, 2008	https://www.r-project.org/
Rstudio	RStudio Team, 2020	https://posit.co/
Tidyverse (R package)	Wickham et al., 2019	https://www.tidyverse.org/
scater (R package)	McCarthy et al., 2017	http://bioconductor.org/packages/release/bioc/html/scater.html

scran (R package)	Lun et al., 2016	https://bioconductor.org/packages/release/bioc/html/scran.html
scuttle (R package)	McCarthy et al., 2017	https://bioconductor.org/packages/release/bioc/html/scuttle.html
patchwork (R package)	Pedersen et al., 2022	https://cran.r-project.org/package=patchwork
bluster (R package)	Lun et al., 2022	https://bioconductor.org/packages/release/bioc/html/bluster.html
batchelor (R package)	Haghverdi et al., 2018	https://bioconductor.org/packages/release/bioc/html/batchelor.html
ggplotlify (R package)	Yu et al., 2021	https://cran.r-project.org/web/packages/ggplotify/index.html
cowplot (R package)	Wilke C, 2020	https://CRAN.R-project.org/package=cowplot
pheatmap (R package)	Kolde K, 2019	https://cran.r-project.org/web/packages/pheatmap/index.html
randomcoloR (R package)	Ammar R, 2019	https://cran.r-project.org/web/packages/randomcoloR/index.html
edgeR (R package)	Robinson et al., 2010; McCarthy et al., 2012; Chen et al., 2016	https://bioconductor.org/packages/release/bioc/html/edgeR.html
DropletUtils (R package)	Lun et al., 2019; Griffiths et al., 2018	https://bioconductor.org/packages/release/bioc/html/DropletUtils.html
Seurat V4 (R package)	Hao et al., 2021	https://satijalab.org/seurat/
basilisk (R package)	Lun et al., 2022	https://www.bioconductor.org/packages/release/bioc/html/basilisk.html
reticulate (R package)	Ushey et al., 2022	https://rstudio.github.io/reticulate/
MOFA2 (R pacakage)	Argelaguet et al., 2018; Argelaguet et al., 2020	https://biofam.github.io/MOFA2/
circlize (R package)	Gu et al., 2022	https://cran.r-project.org/web/packages/circlize/index.html
ComplexHeatmap (R package)	Gu et al., 2022	https://bioconductor.org/packages/release/bioc/html/ComplexHeatmap.html
fgsea (R package)	Korotkevich et al., 2019	https://bioconductor.org/packages/release/bioc/html/fgsea.html
miloR (R package)	Morgan et al., 2022	https://www.bioconductor.org/packages/release/bioc/html/miloR.html
monocle (R package)	Trapnell et al., 2014; Qiu et al., (2017); Qiu et al., (2017)	https://www.bioconductor.org/packages/release/bioc/html/monocle.html
clusterProfiler (R package)	Wu et al., 2021; Yu et al., 2012	https://bioconductor.org/packages/release/bioc/html/clusterProfiler.html
org.Hs.eg.db (R package)	Carlson et al., 2019	https://bioconductor.org/packages/release/data/annotation/html/org.Hs.eg.db.html
msigdbr (R package)	Bhuva et al., 2022	https://bioconductor.org/packages/release/data/experiment/html/msigdb.html
ggpubr (R package)	Kassambara et al., 2023	https://cran.r-project.org/web/packages/ggpubr/index.html
GSVA (R package)	Hänzelmann et al., 2013	https://www.bioconductor.org/packages/release/bioc/html/GSVA.html
tableone (R package)	Yoshida et al., 2022	https://CRAN.R-project.org/package=tableone
Analysis code	https://github.com/jenssle/CITEseq_MM_CoV_Vac	N/A
Other		

BD FACS Celesta Cell Analyzer	BD Biosciences	Cat#660346
DxFlex Flow Cytometer	Beckman Coulter	Cat#C02846
BD FACS Aria Fusion III Cell sorter	BD Biosciences	Cat#657590
BD Rhapsody Single-Cell Analysis System: Scanner & Express Instrument	BD Biosciences	Cat#633701 & Cat#633702
Disposable hemocytometer	Incyto	Cat# DHC-N01-5
Qubit 4 Fluorometer	Thermo Fisher Scientific	Cat#Q33238
4150 TapeStation System	Agilent	Cat#G2992AA
Illumina NextSeq 2000	Illumina	Cat#20038897
Abbott Alinity i platform	Abbott	AI 03932

Tab. S7: Used materials and resources

Includes all used materials and other resources for this study.
